# Supplementary material for: Nearly complete structure of bacteriophage DT57C reveals architecture of head-to-tail interface and lateral tail fibers
Source: Nat Commun. 2023 Dec 11;14:8205. doi: 10.1038/s41467-023-43824-9 (PMC10713586; doi:10.1038/s41467-023-43824-9)
Supplement: Supplementary file 1 — Supplementary Information [file 41467_2023_43824_MOESM1_ESM.pdf]

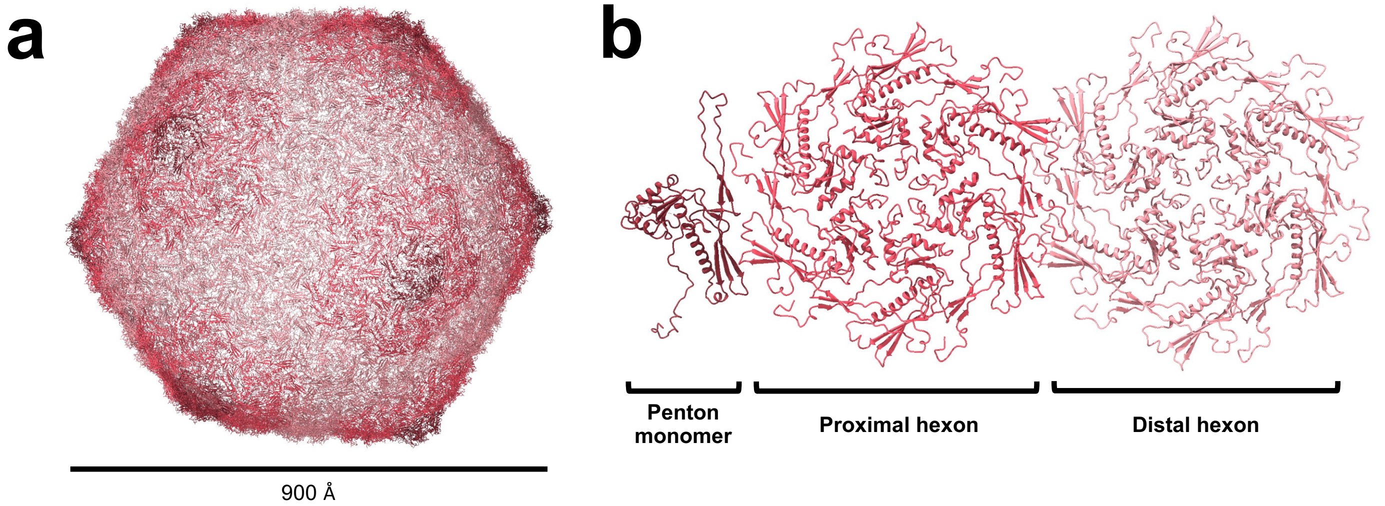


**Supplementary Figure 1. Capsid of the DT57C phage. a,** Expanded capsid of the DT57C phage without DCP. The capsid has a diameter of approximately 900 Å. **b,** Asymmetric unit of the capsid of the DT57C phage. Excluding the DCP, the asymmetric unit contains 13 MCP copies: one contributing to the formation of pentons at icosahedral verteces (dark red), 6 forming a hexon directly adjacent to the pentons (proximal hexon; red) and 6 forming another hexon not adjacent to the penton (distal hexon; pink).

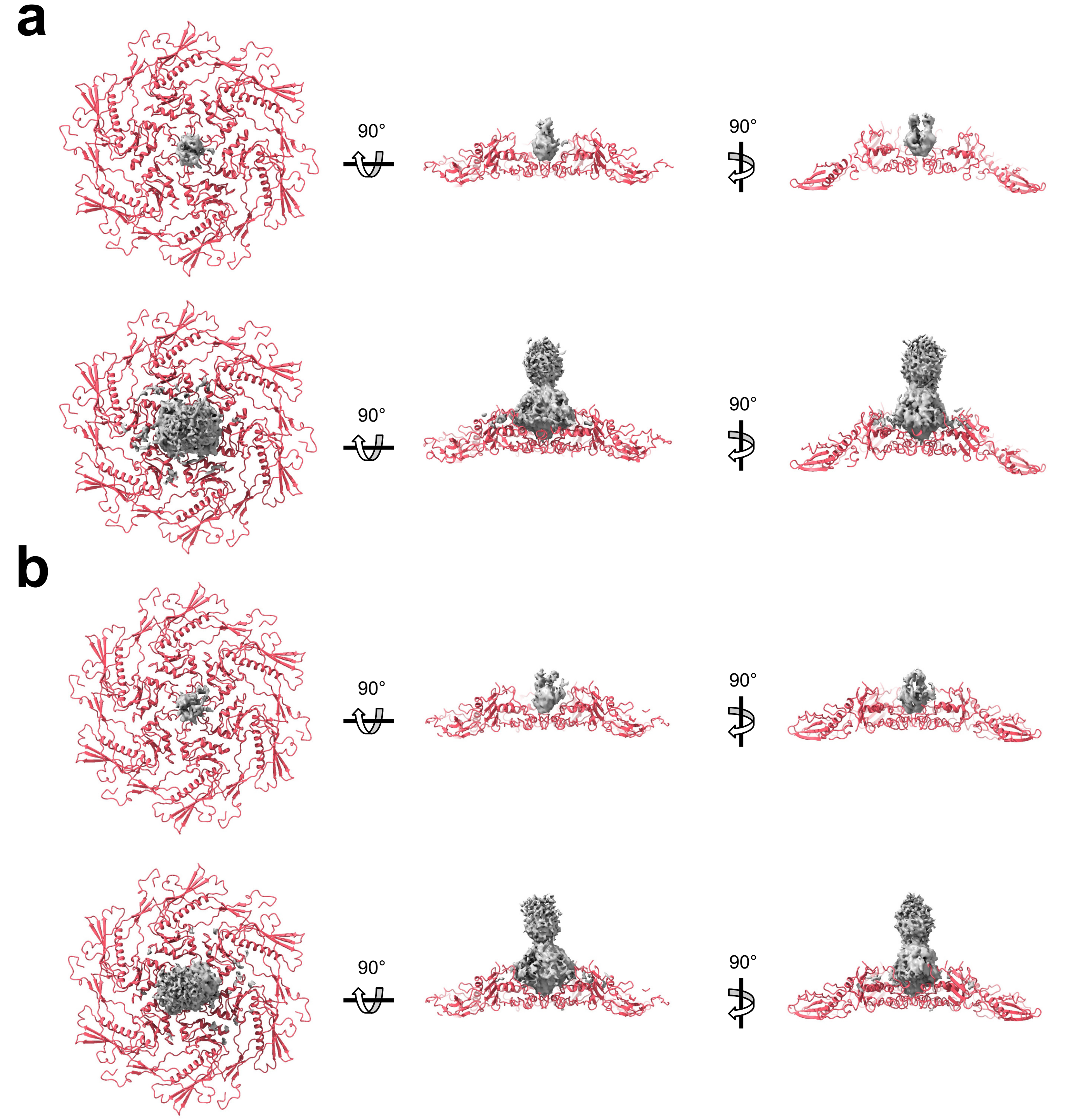


**Supplementary Figure 2. Decoration protein of the DT57C phage. a,** Density left in the center of the proximal hexon after subtracting the MCP from the map at 4σ (top) and 1.5σ (bottom). The density visible at high threshold corresponds to the portion of the N-terminal domain directly interacting with the hexon, while the first Ig-like domain of the DCP only becomes visible at lower thresholds due to its flexible orientation with respect to the hexon. The density corresponding to the N-terminal domain shows 2-fold-symmetry-like features, indicating that the DCP binds in one of 2 preferred orientations to the center of hexons, and not randomly in any of the 6 *a priori* equivalent possible positions. **b,** As in **a** but for the distal hexon. The 2-fold-symmetry-like features could also be alternatively explained by the binding of a dimer of decoration proteins to the center of each hexamer. Nevertheless, previous studies indicate that a single copy of the decoration protein binds to each hexamer[13]. Furthermore, calculation of the protein mass enclosed by the density corresponding to the first Ig-like domain using a reasonable threshold (3.0σ) and assuming a protein density of 0.83 Da·$Å^{-3}$ resulted in a value of approximately 6,000 Da. This is close to the expected value of around 7,500 Da for the first Ig-like domain, and too far from what would be expected for 2 close copies of it, therefore indicating that it is likely that a single decoration protein copy binds to each individual hexon.

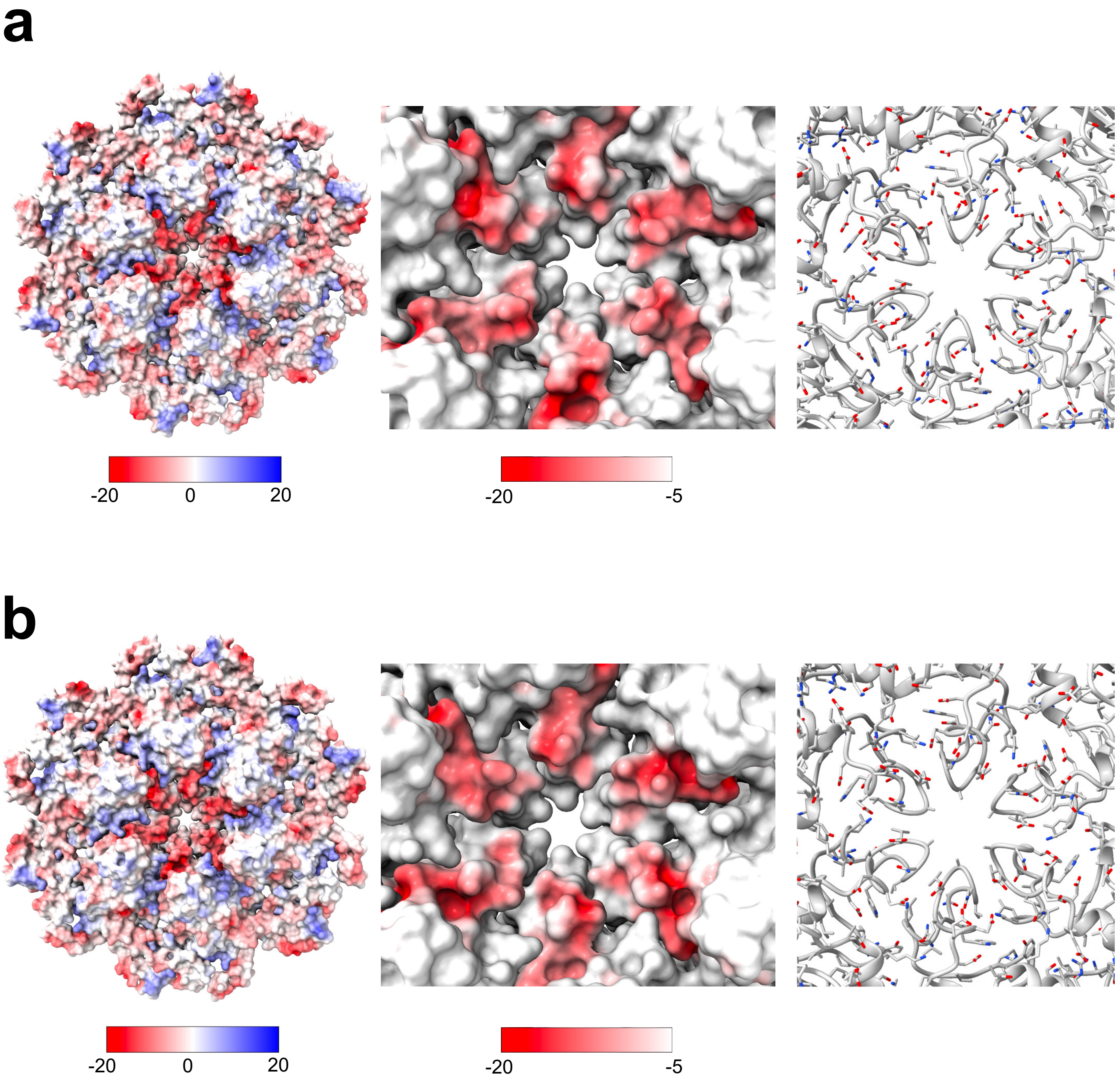


**Supplementary Figure 3. Electrostatic potential of the DCP binding site. a,** Coulombic electrostatic potential for the proximal hexon. The center of the hexon has a high negative electrostatic potential. Furthermore, the electrostatic potential and surface shape is not 6-fold symmetric around the center of the hexon. Such absence of perfect 6-fold symmetry around the center of each hexon may be the cause for the DCP not binding randomly in any of the a priori equivalent six binding modes (as would be expected if each hexon displayed true 6-fold symmetry around its center), having instead two preferred binding modes. **b,** As in **a** but for the distal hexon. Electrostatic potential units are kcal(mol·*e*) at 298 K.

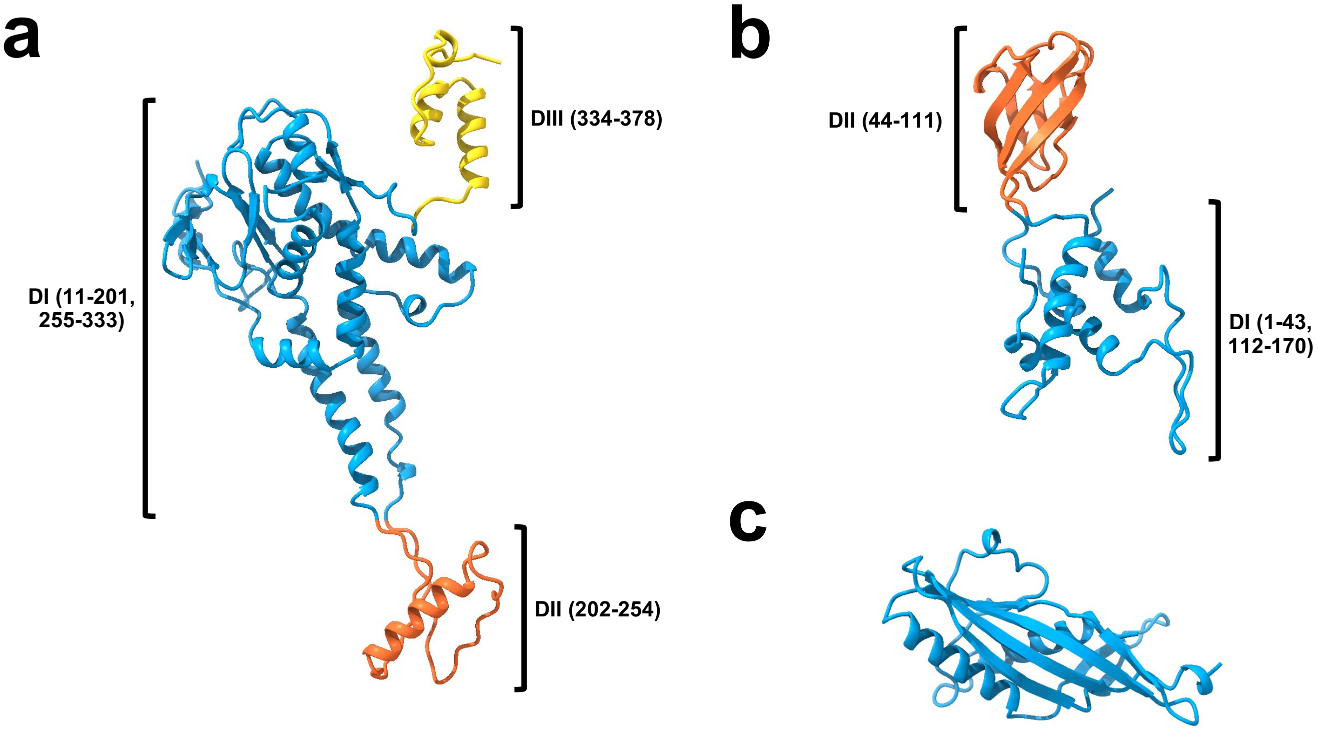


**Supplementary Figure 4. Structure of the proteins composing the neck area. a**, Portal protein (PrtP). **b**, Head completion protein (HCP). **c**, Tail completion protein (TCP).

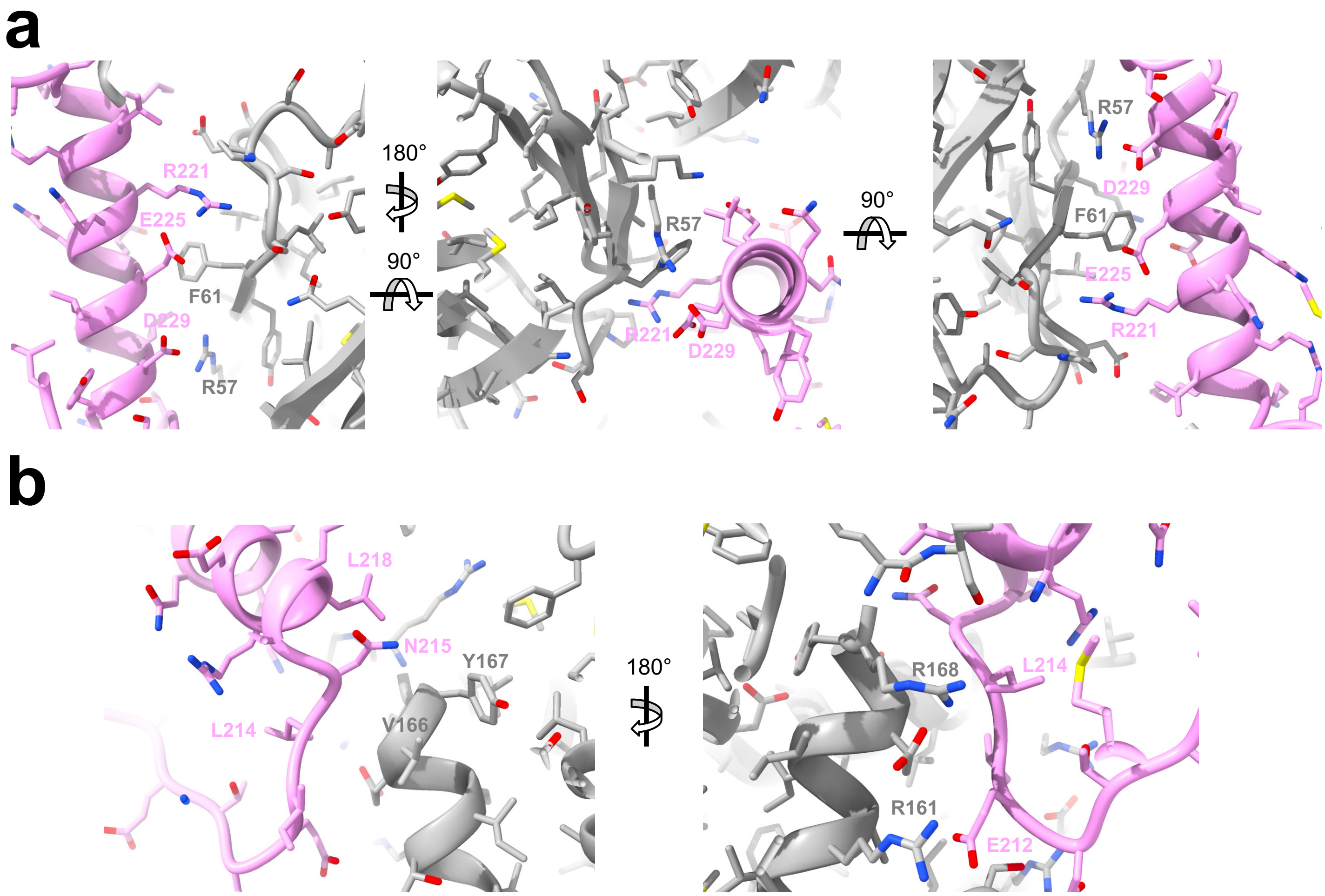


**Supplementary Figure 5. Interaction between PrtP and HCP. a,** First interaction surface, involving the α-helix formed by residues 216-230 of PrtP (pink) and a small β-sheet of HCP (grey) including residues 57-65 and 89-95. **b**, Second interaction surface, involving a loop region of PrtP (residues 211-215), and a loop (residues 153-156) and an α-helix (158-167) of HCP.

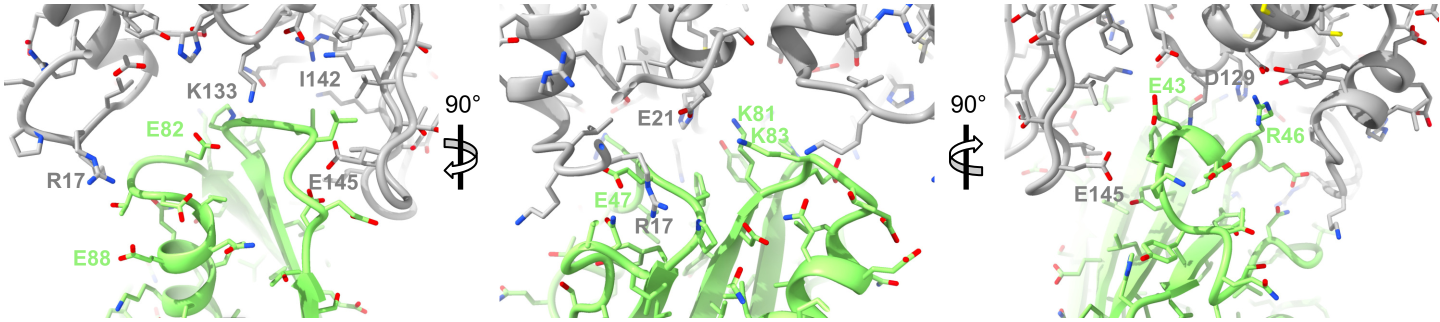


**Supplementary Figure 6. Interaction between HCP and TCP.** The interaction between the TCP hexameric ring and the HCP dodecameric ring is mediated by the DI of two adjacent HCP monomers (through loops comprising residues with residues 13-17 and 132-138, and β-hairpin formed by residues 139-148), which contact the top part of a single TCP monomer (loops comprising residues 41-49, 81-86 and 134-141).

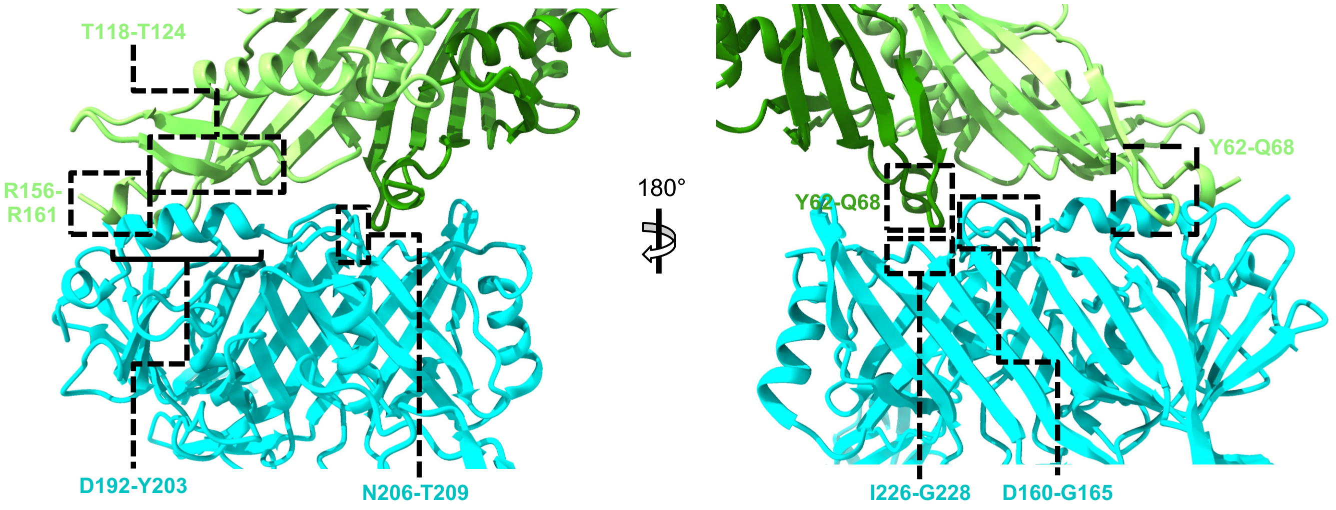


**Supplementary Figure 7. Interaction between TCP and TTP.** Each monomer of the first TTP (cyan) trimeric ring is contacted by two TCP monomers (TCP I – light green; TCP II – dark green). One interaction surface is established between α-helix comprising residues 192-203 and loops comprising residues 29-34 and 7-10 from TTP, and loop with residues 62-68, a small α-helix formed by residues 156-161 and a β-strand involving residues 118-124 from TCP I. The second interaction area is established between loops comprising residues 160-165, 206-209 and 226-228 of TTP, and loop formed by residues 62-68 of TCP II. The hexameric TCP ring therefore acts as an adapter between the preceding HCP dodecameric ring and the first trimeric TTP ring of the tail tube.

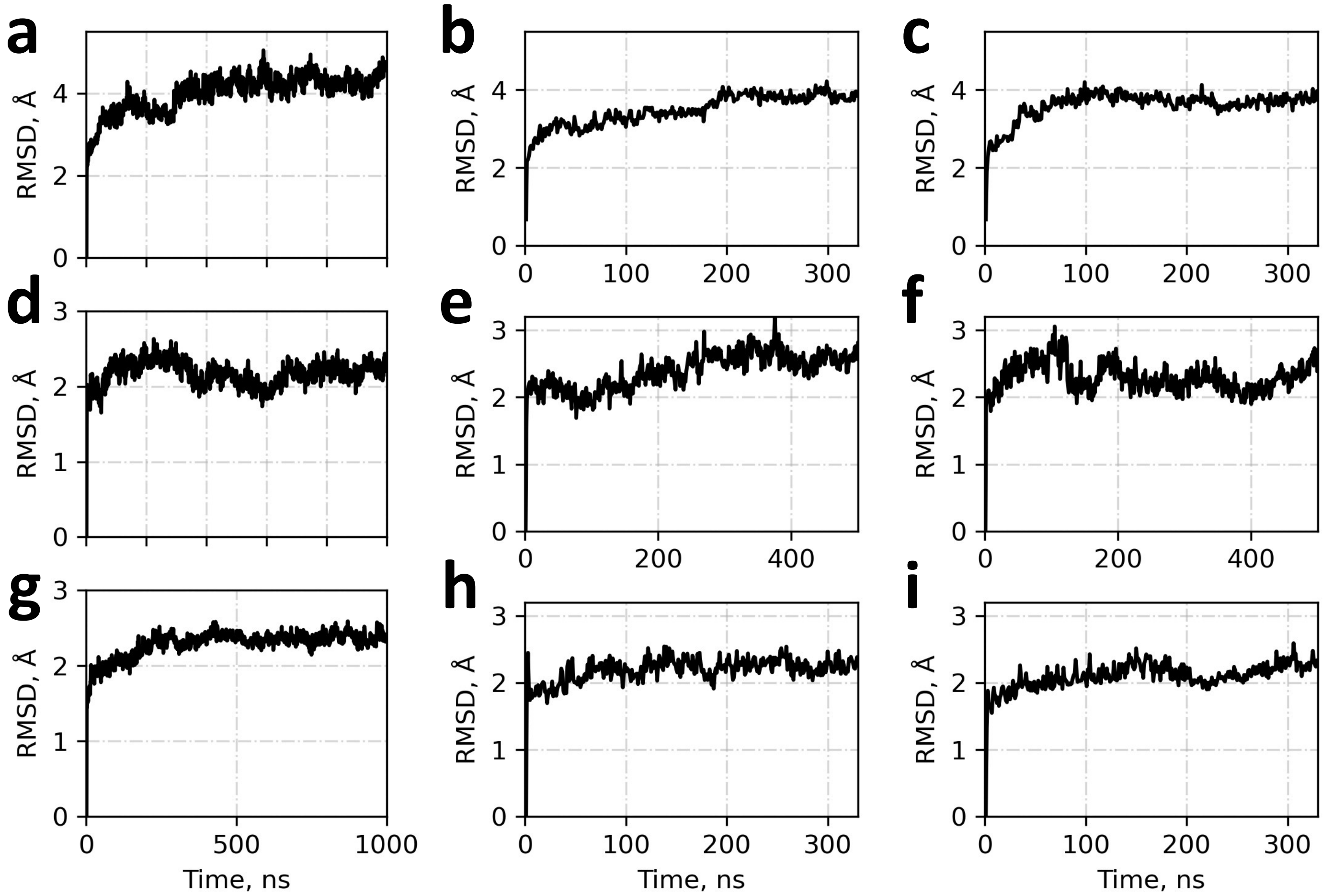


**Supplementary Figure 8. Evolution of root mean square deviations (RMSD) of the backbone atoms of all simulated systems**. **a-c**, LtfA-LtfC-TTMP ring**. d-f,** HCP ring**. g-i,** HCP-TCP ring. For each system, three simulation replicates are shown. Source data are provided as a Source Data file.

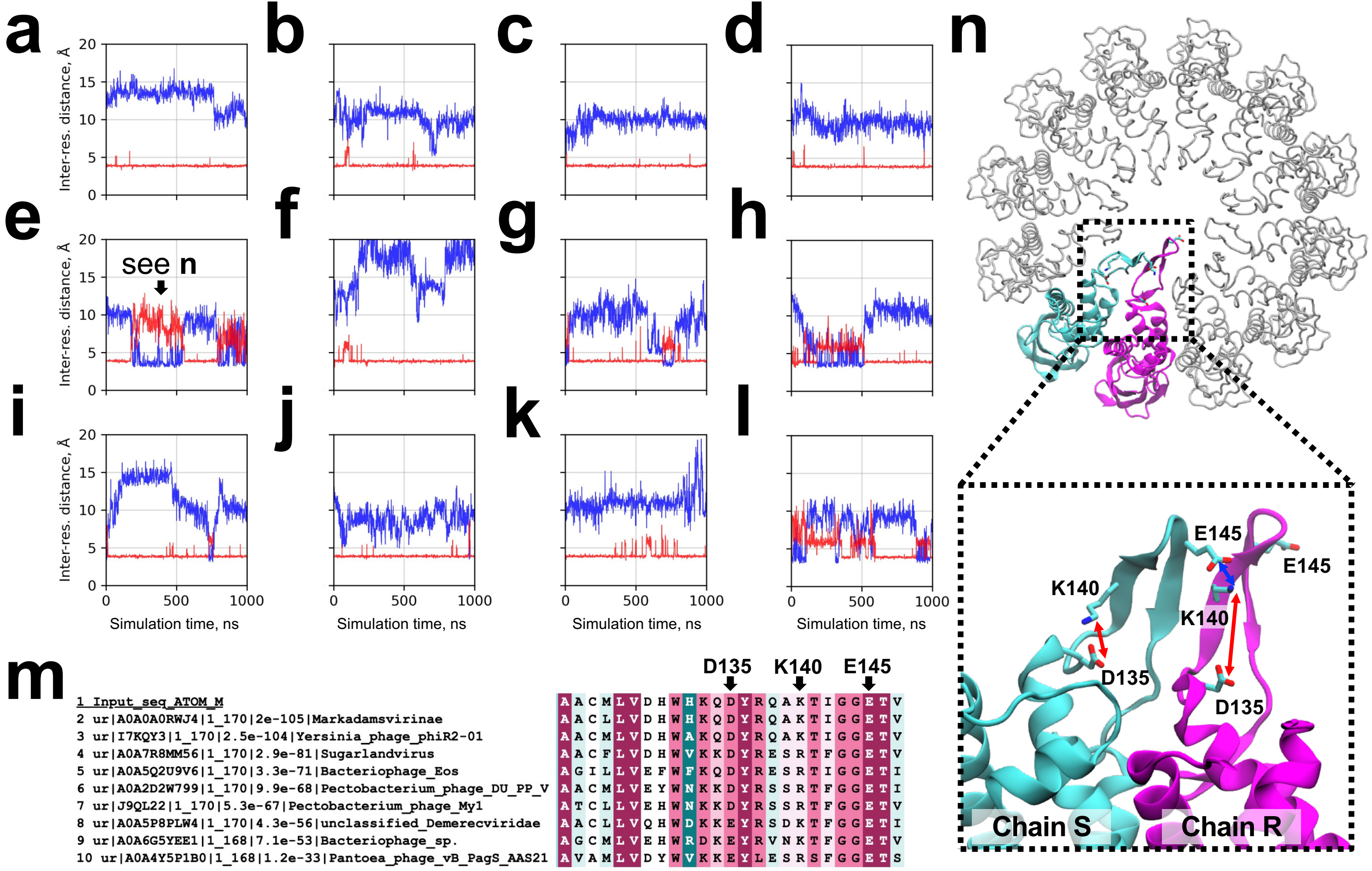


**Supplementary Figure 9. Contacts in HCP protomers in the absence of the TCP ring. a-l,** Inter-residue distances between D135-K140 of each HCP protomer (red) and K140-E145 of neighboring HPC protomers (blue) in the simulation of the HCP ring. Formation of the ionic lock between K140 andE145, which stabilizes the inward-pointed conformation of the β-hairpin of HCP, can be observed in panels **e**, **g**, **h**, **i**, and **l** (regions with the inter-residue distance < 5 Å). **m**, multiple sequence alignment of HCP homologs from Uniref90 database obtained using the Confsurf server. All three positions in MSA, D(E)135, K(R)140, and E145, are conserved. **n**, Ionic bridges between D135-K140 and K140-E145 in two neighboring protomers of HCP. The corresponding trajectory frame is highlighted with a black arrow in panel **e**. Source data for panels **a-l** are provided as a Source Data file.

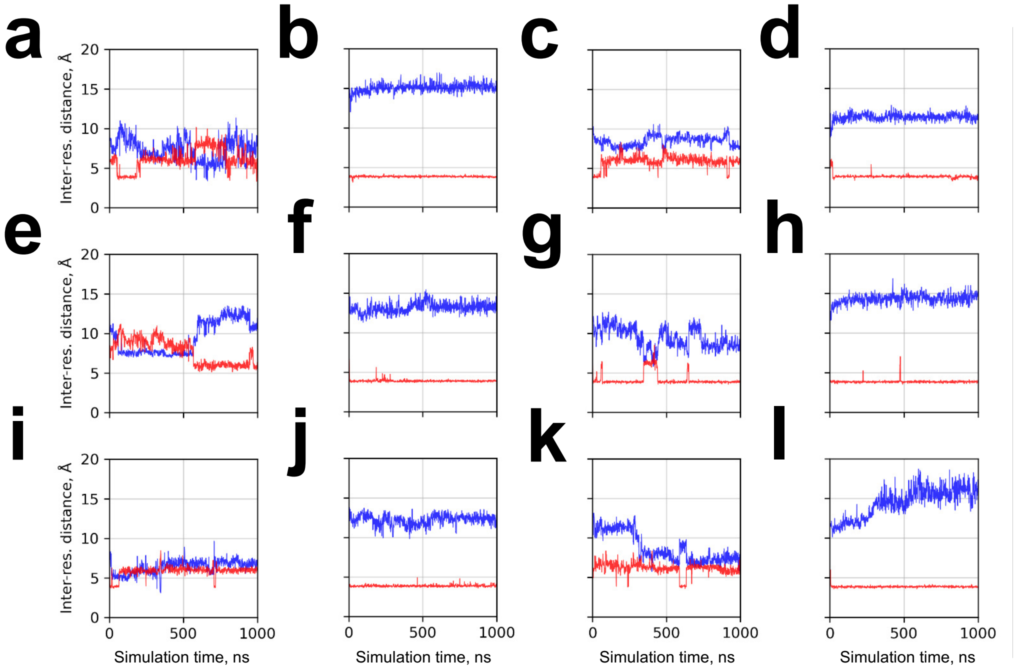


**Supplementary Figure 10. Contacts in HCP protomers in the presence of the TCP ring.** Inter-residue distances between D135-K140 of each HCP protomer (red) and K140-E145 of neighboring HPC protomers (blue) in the simulation of the HCP ring along with the TCP ring are shown. The ionic lock between K140 and E145 (regions with the inter-residue distance < 5 Å) does not form in any of the HCP protomers. Source data are provided as a Source Data file.

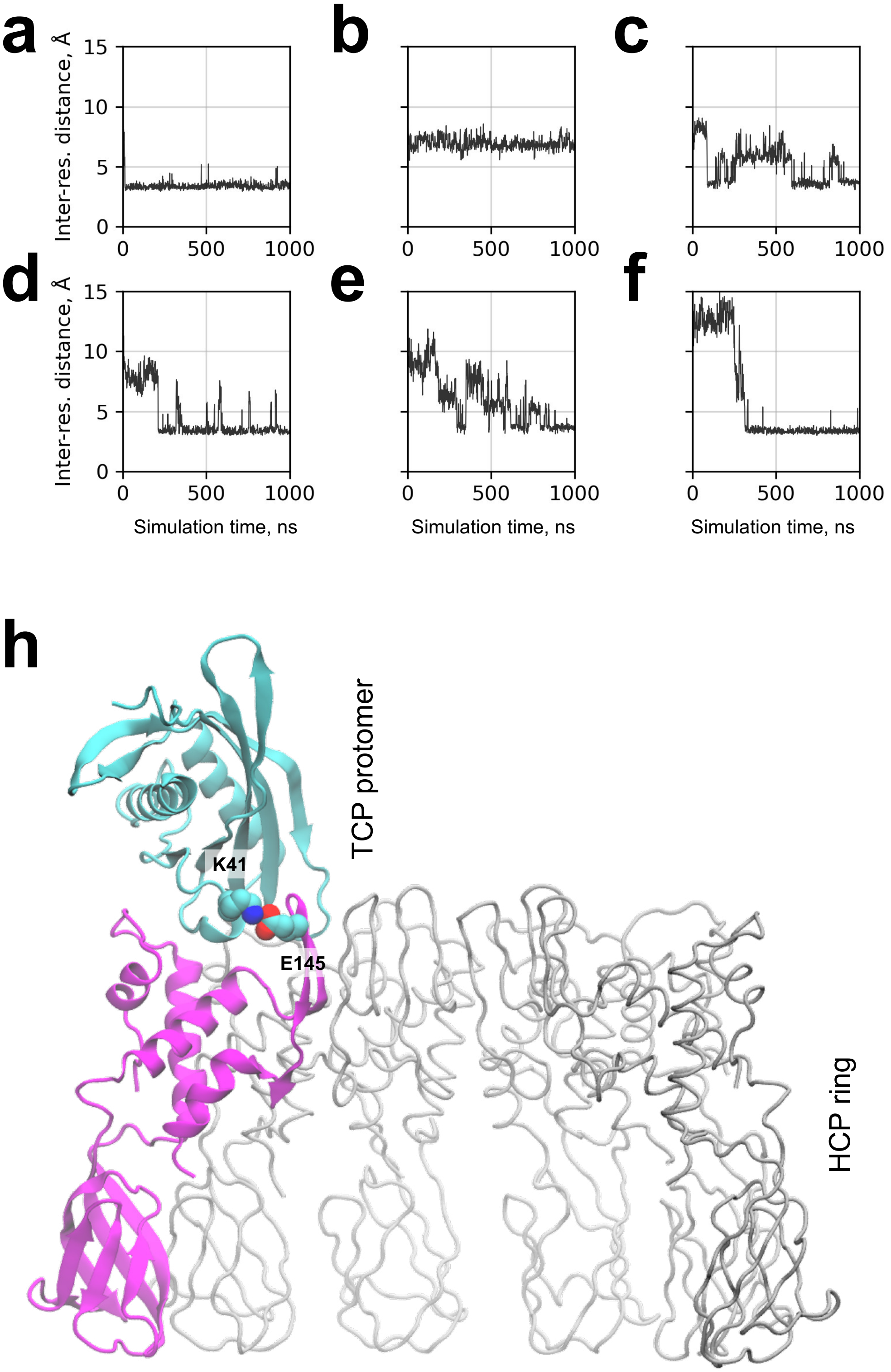


**Supplementary Figure 11. Contacts between HCP and TCP protomers. a-f,** Inter-residue distances between E145 of HCP and K41 of adjacent TCP promomer. Due to the mismatch in the number of protomers in the HCP and TCP rings, only six such contacts can potentially form. **m**, Example of the ionic bridge between E145 and K41 observed in the molecular dynamics sumulation of the HCP-TCP system. This ionic bridge replaces the potential ionic interaction between E145 and K140 and presumably stabilizes the open conformation of the pore. Source data for panels **a-f** are provided as a Source Data file.

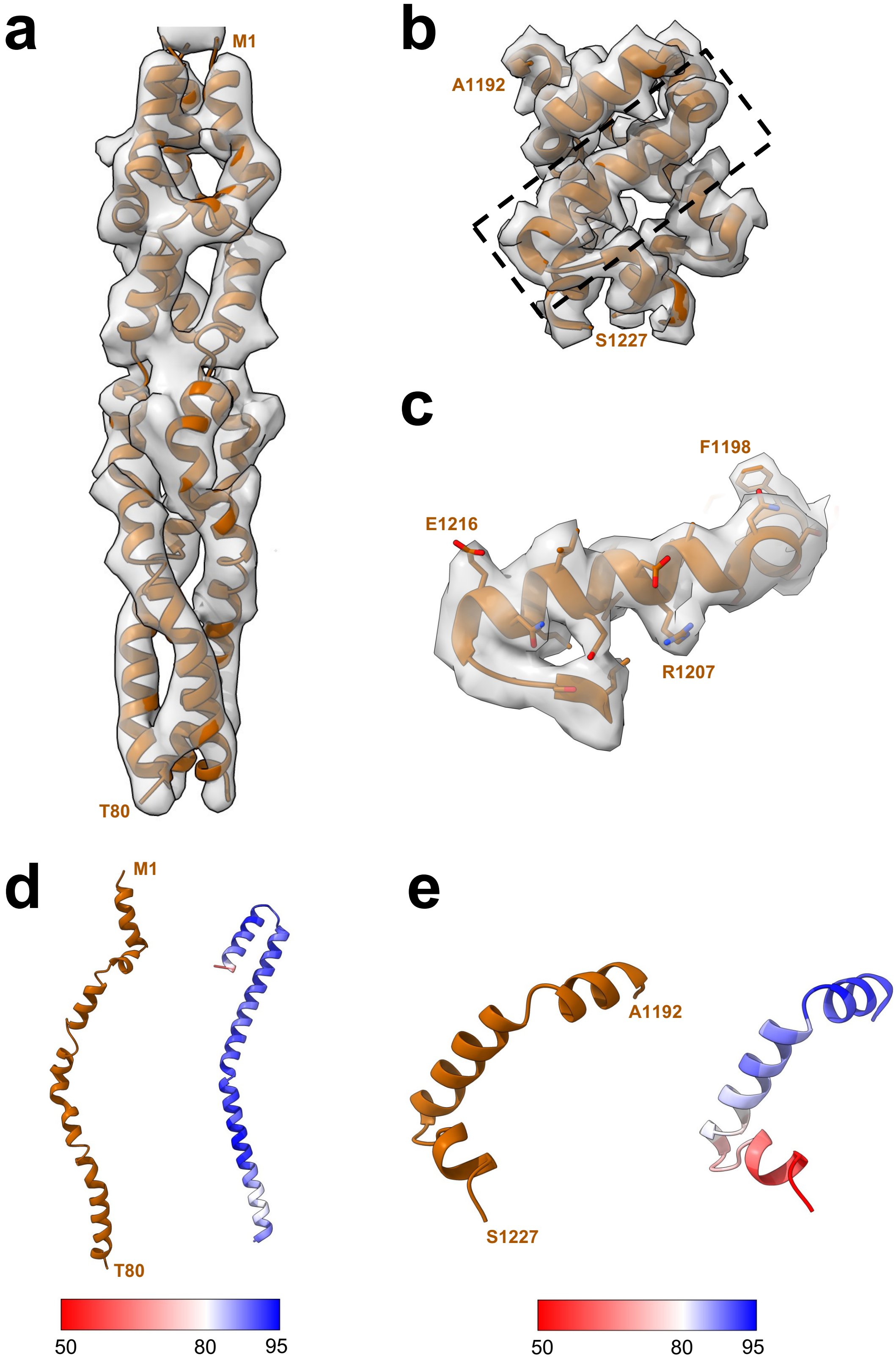


**Supplementary Figure 12. Fitting of TMP regions. a,** N-terminal region (80 residues), located in the neck area. **b**, C-terminal region (residues 1192-1227), located in the tail tip complex area. **c**, Example of fitting of side chains in the TMP C-terminal region highlighted by the box in panel **b**. **d**, Comparison of model built for the initial 80 residues of the N-terminus of the TMP (left) and the AlphaFold prediction for the same region (right), colored by confidence of the prediction. **e**, As in **d** but for the C-terminal region (residues 1192-1227) of the TMP. Confidence of the prediction is displayed in %.

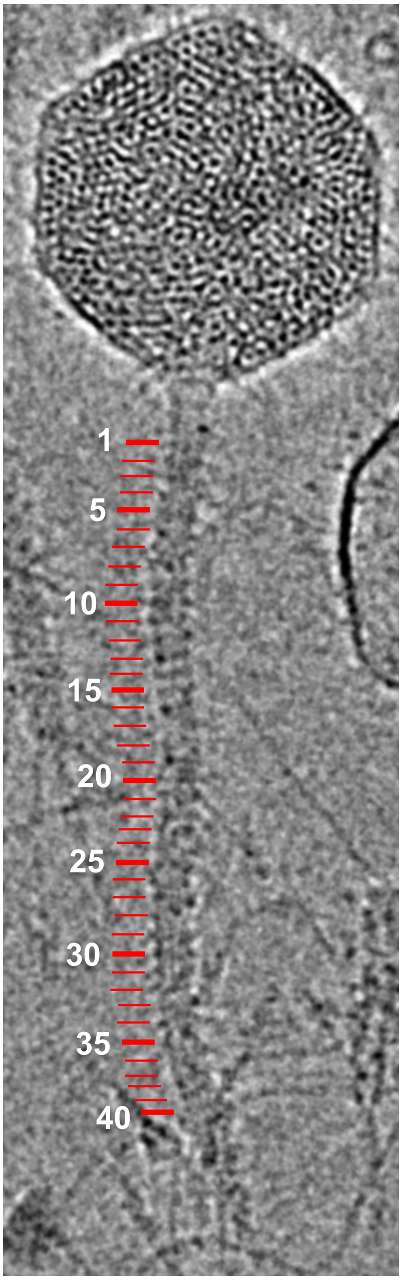


**Supplementary Figure 13. Length of the DT57C tail.** Analysis of individual DT57C particles revealed the presence of 40 TTP rings, similar to what had been previously described for the T5 phage. This is in accordance with the very similar length of the TMP protein of both phages.

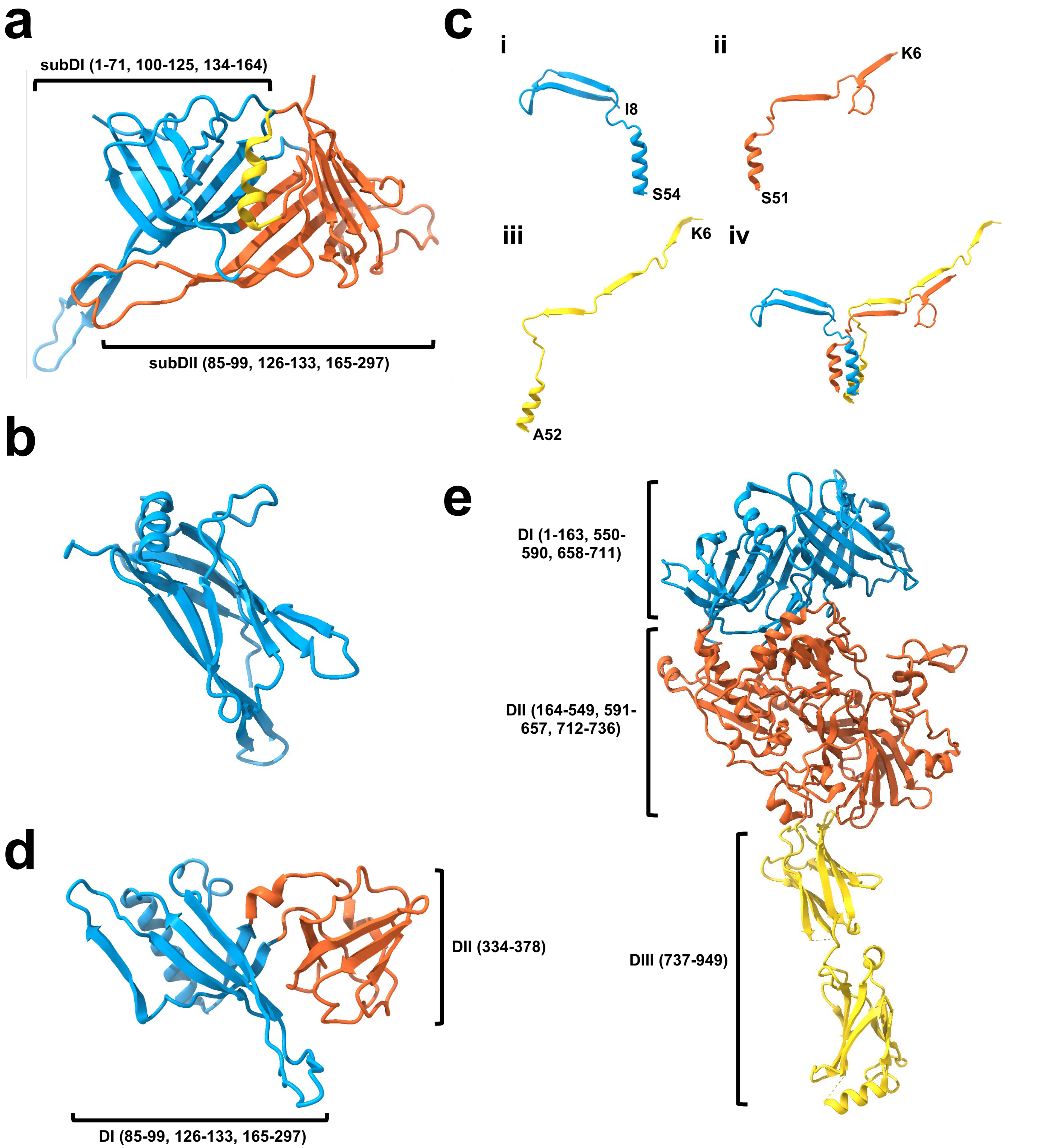


**Supplementary Figure 14. Structure of the proteins composing the baseplate area. a**, Tail tip middle protein (TTMP). **b**, Lateral tail fiber C (LtfC). **c**, Lateral tail fiber A (LtfA). i-iii, conformation of each of the monomers composing an LtfA trimer. iv, LtfA trimer. **d**, Distal tail protein (Dit). **e**, Baseplate hub protein (BHP).

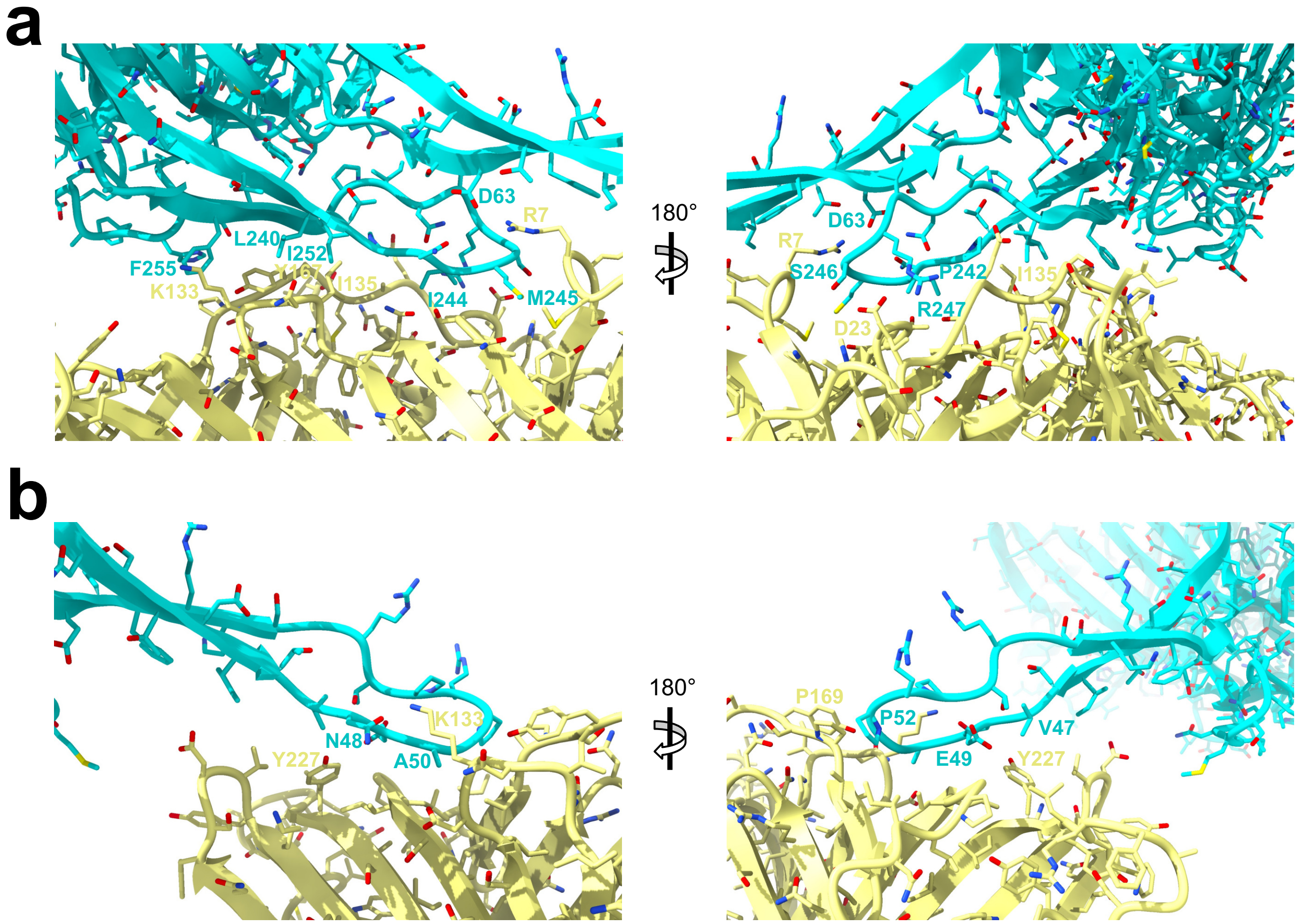


**Supplementary Figure 15. Interaction between TTP and TTMP.** Each monomer of the last TTP ring (cyan) contacts 2 TTMP monomers (light yellow). **a,** First interaction surface, established between β-hairprin comprising residues 237-256 of TTP, and loop regions comprised by residues 6-8, 23-27, 132-135 and 161-169 of a TTMP monomer. **b**, Second interaction surface, established between β-hairprin comprising residues 237-256 of TTP, and loop/β-strand regions comprised by residues 131-133, 169-171, 180-183, 226-228 and 270-274 of another TTMP monomer.

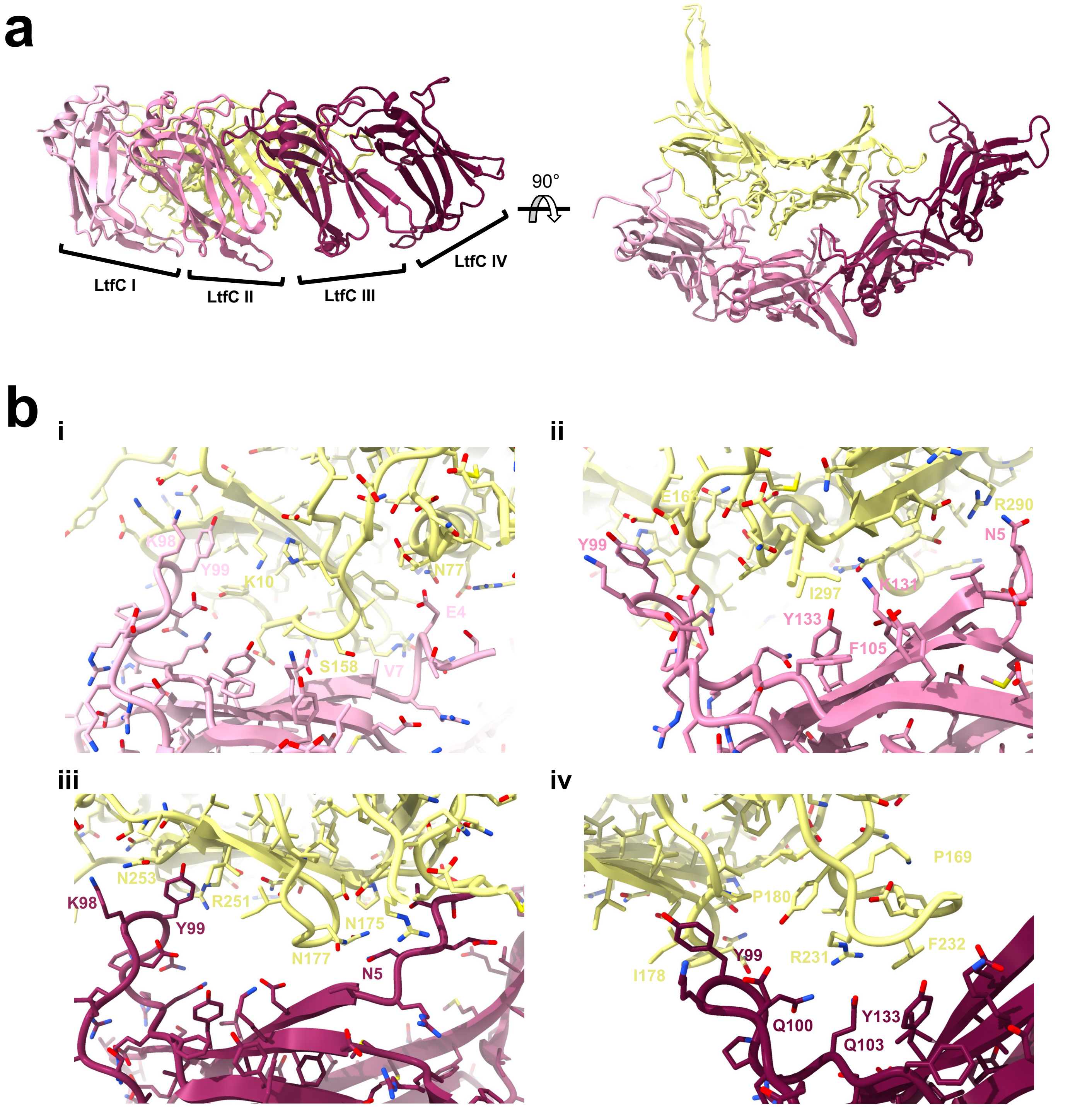


**Supplementary Figure 16. Interaction between LtfC and TTMP. a,** Overview of the interaction between the asymmetric units of LtfC (shades of pink/maroon) and TTMP (light yellow) in the baseplate. Each TTMP monomer contacts four LtfC monomers through its outer face. **b**, Detail of the interaction between the TTMP monomer and each LtfC monomer. *i*, The interaction surface is established between the areas of LtfC I comprising residues 3-11, 97-101 and 130-134, and residues 73-77, and regions of TTMP comprising residues 8-10, 73-77, 110-112 and 153-159. *ii*, The interaction surface is established between the areas of LtfC II comprising residues 97-102, 105-108 and 131-133, and regions of TTMP comprising residues 158-163 and 295-297. *iii*, The interaction surface is established between the areas of LtfC III comprising residues 2-9 and 98-100, and regions of TTMP comprising residues 170-172, 175-177 and 251-253. *iv*, The interaction surface is established between the areas of LtfC IV comprising residues 9-11, 98-103 and 131-133, and regions of TTMP comprising residues 178-181 and 231-234.

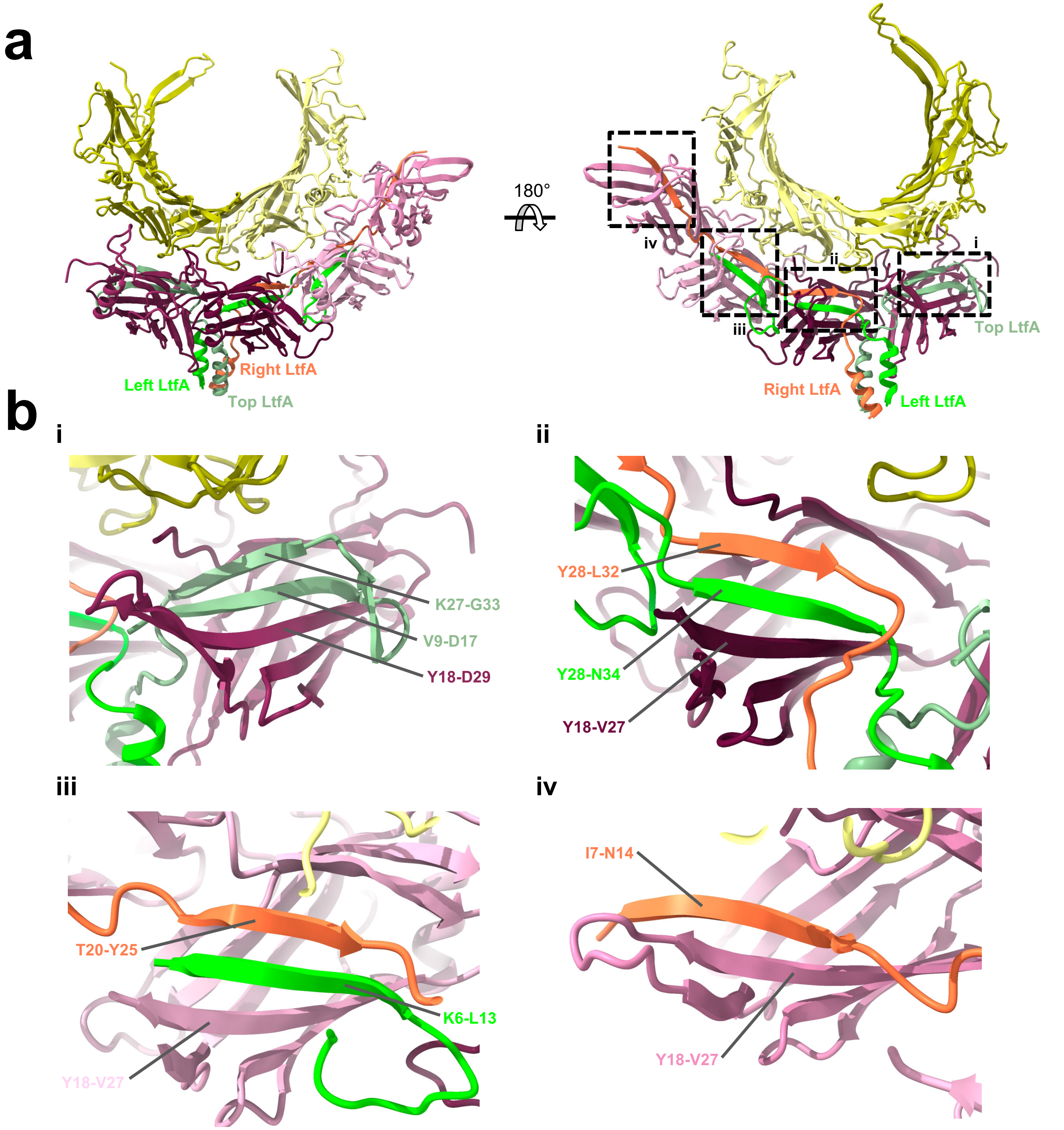


**Supplementary Figure 17. Interaction between LtfA and LtfC. a,** Overview of an LtfA trimer sandwiched between four LtfC monomers and two TTMP monomers. **b**, Extension of LtfC β-sheets through the contribution of additional strands by LtfA monomers.

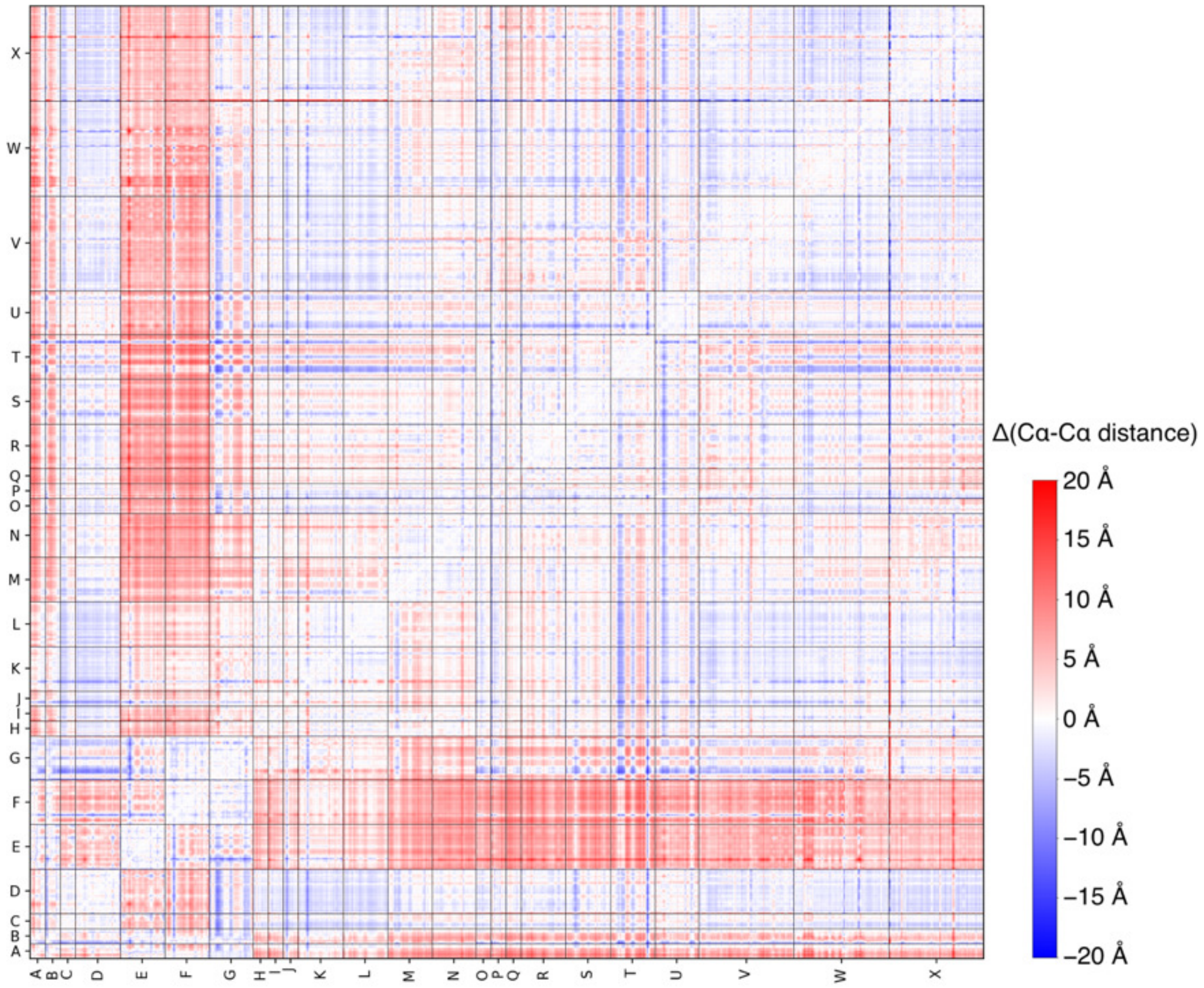


**Supplementary Figure 18. Difference between the inter-residue $C_\alpha$-$C_\alpha$ distances in the initial model and the final snapshot of the molecular dynamics trajectory of LtfA-LtfC-TTMP.** Positive/negative values correspond to residue pairs which appear more distant/closer upon the simulation. Chains A+B+C, H+I+J, and O+P+Q correspond to LtfA trimers; D,E,F,G, K, L, M, N, R, S, T, U to the LtfC ring; V, W, and X to the TTMP trimer. Source data are provided as a Source Data file.

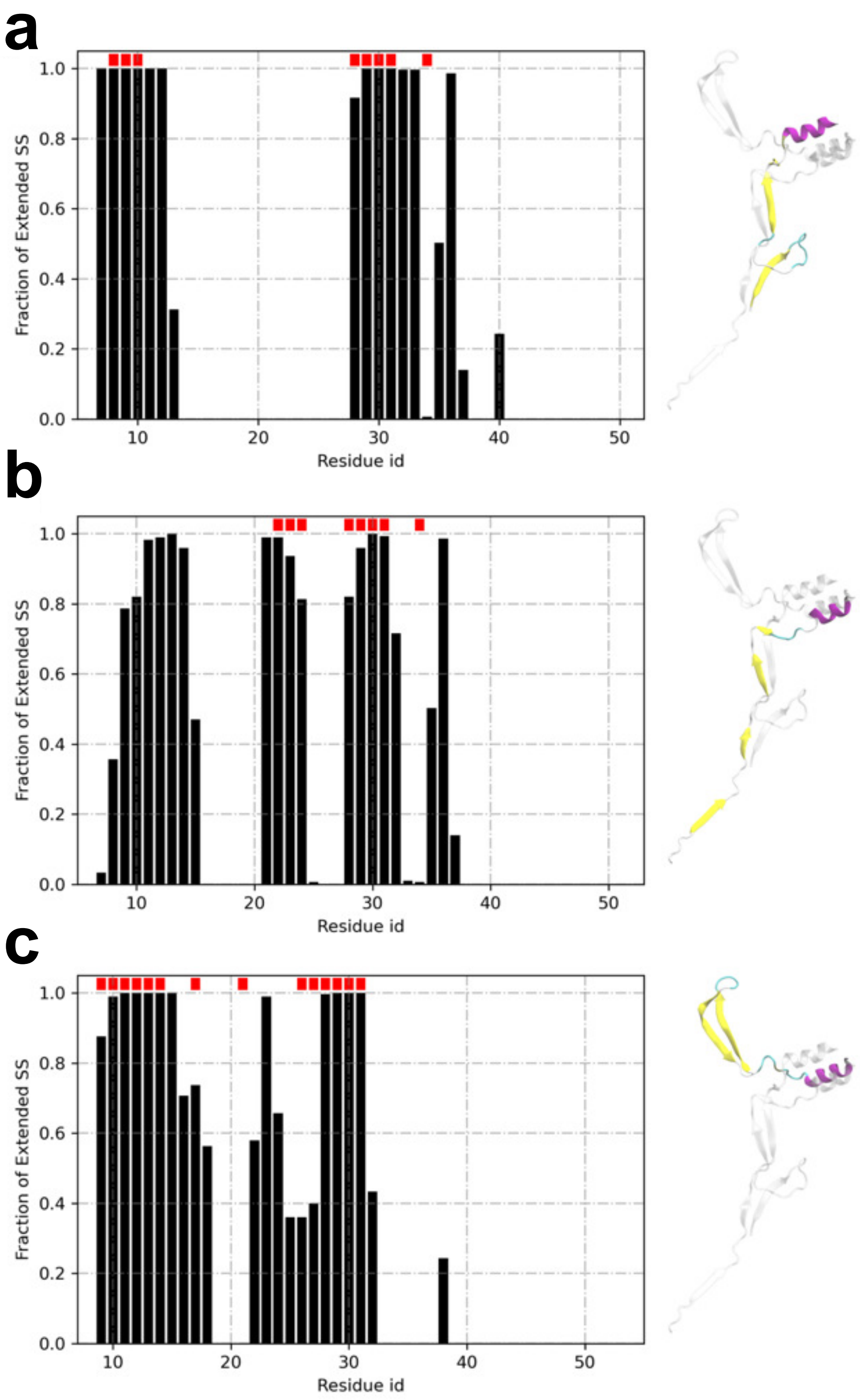


**Supplementary Figure 19. Propensity of the LtfA residues to form extended secondary structure (β-sheets or isolated β-bridges).** Panels **a-c** correspond to the three different monomers of the LtfA trimer (shown along the corresponding histograms). Values for each monomer are averaged over three LtfA trimers. The red bars at the top of the histograms indicate the residues possessing the extended conformation in the initial model. Source data are provided as a Source Data file.

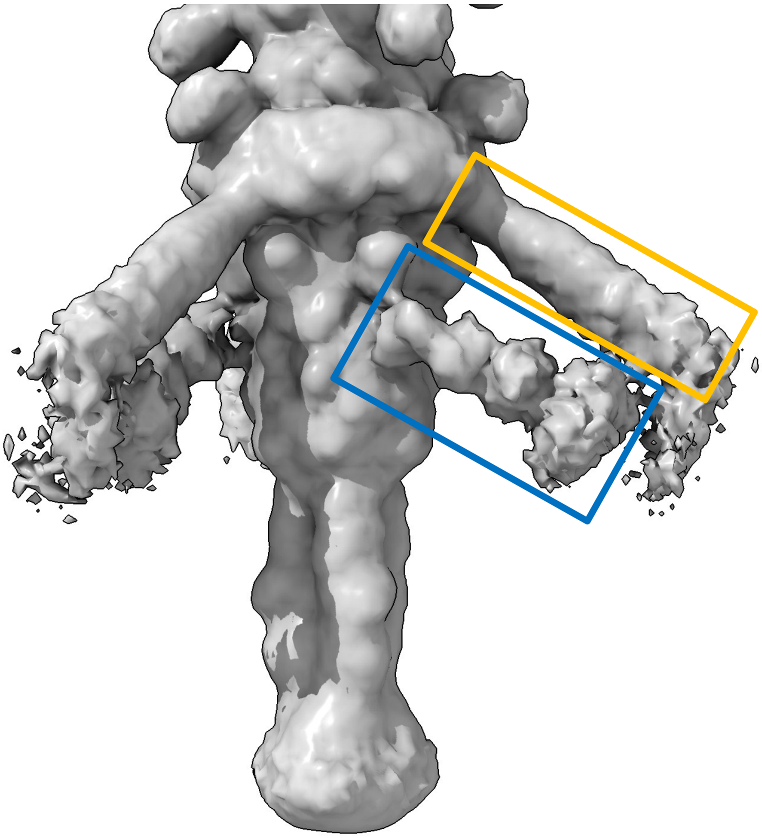

**Supplementary Figure 20. Weak density protruding from TTHP.** Additional weaker density was observed protruding from TTHP when the model was contoured at a low threshold (blue box), which contacted the density corresponding to LtfA (yellow box). We assign the density to likely correspond to the LtfB component of the lateral tail fibers.

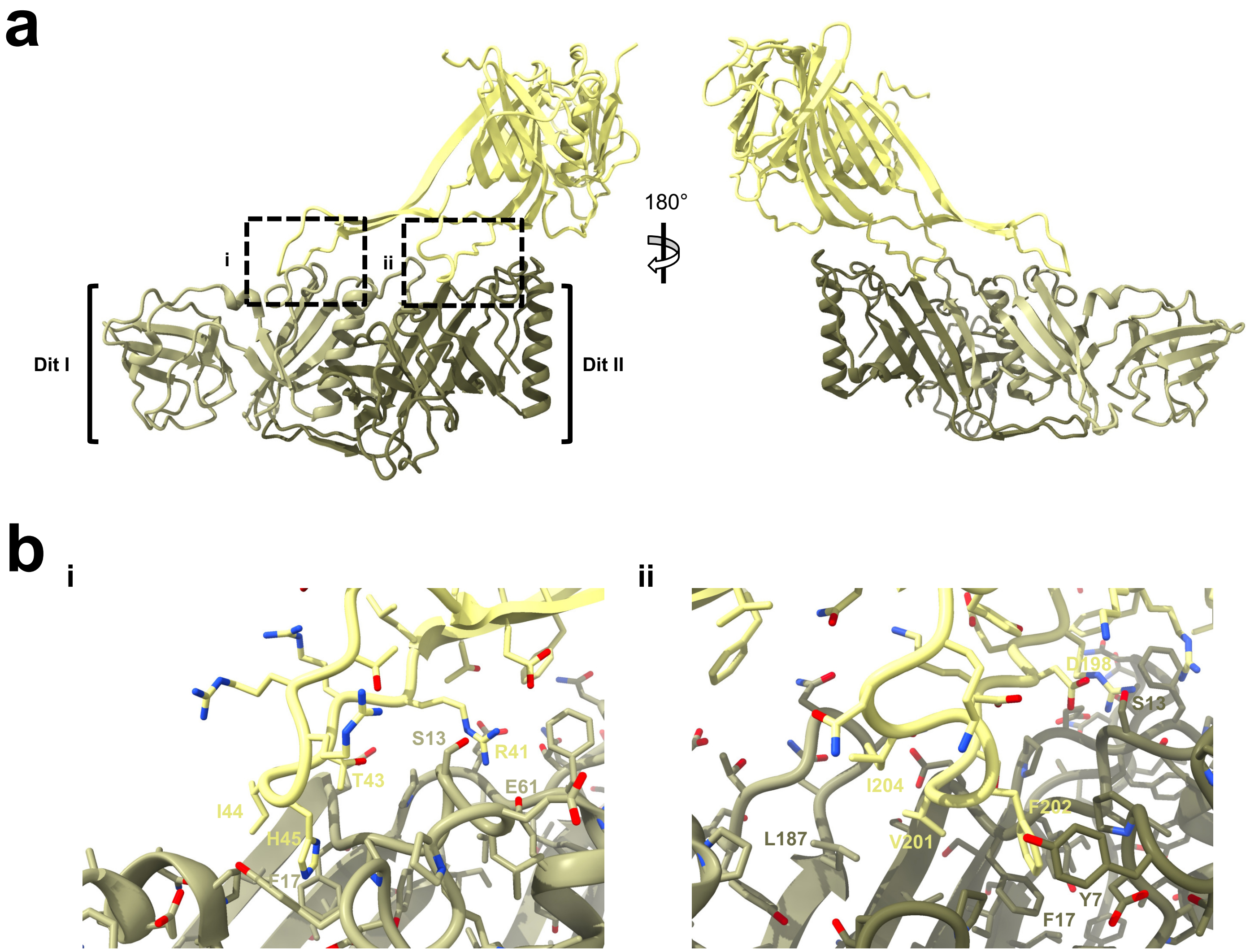


**Supplementary Figure 21. Interaction between TTMP and Dit. a,** Overview of the interaction between the asymmetric units of TTMP and Dit in the baseplate. Each TTMP monomer (light yellow) contacts four two Dit monomers (dark yellow shades) through its protruding β-hairpins. **b**, Detail of the two interaction areas between the TTMP monomer and each Dit monomer. *i*, The interaction surface is established between the β-hairpin of TTMP comprising residues 37-57, and regions of Dit I comprising residues 13-19, 55-58 and 190-192. *ii*, The interaction surface is established between the β-hairpin of TTMP comprising residues 195-217, and regions of Dit II comprising residues 6-7, 11-19, 55-61, 82-84 and 190-192, as well as residues 188-192 from Dit I.

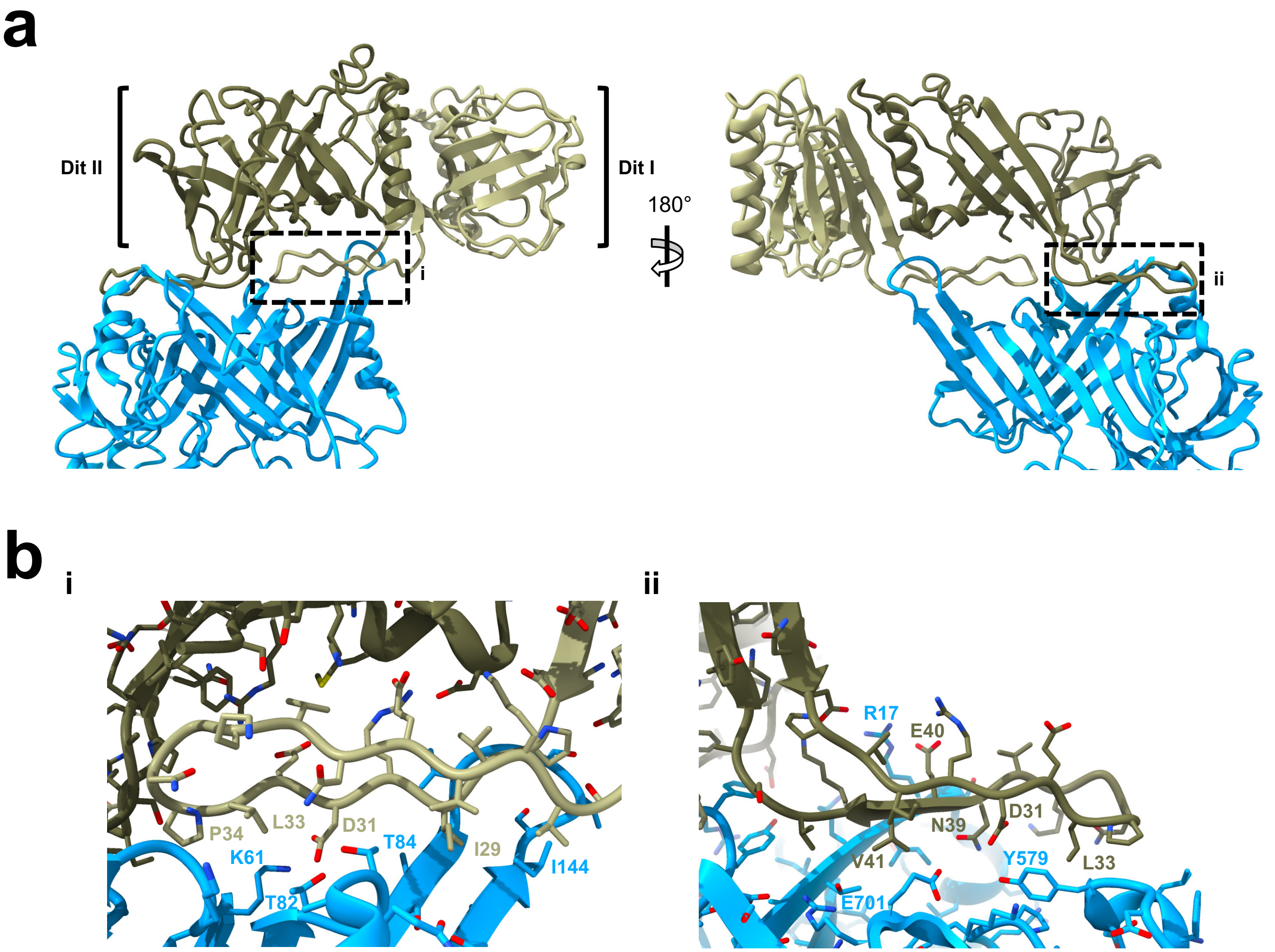


**Supplementary Figure 22. Interaction between Dit and TTHP. a,** Overview of the interaction between the asymmetric units of Dit and TTHP in the baseplate. Two Dit monomers (dark yellow shades) contact the top part of each TTHP monomer (blue). **b**, Detail of the two interaction areas between the Dit monomers and each TTHP monomer. *i*, The interaction surface is established between the regions of TTHP comprising residues 57-61, 82-84, 144-151, and the β-hairpin of Dit I comprising residues 26-45. *ii*, The interaction surface is established between the regions of TTHP comprising residues 16-19, 576-580 and 700-703, and the β-hairpin of Dit II comprising residues 26-45.

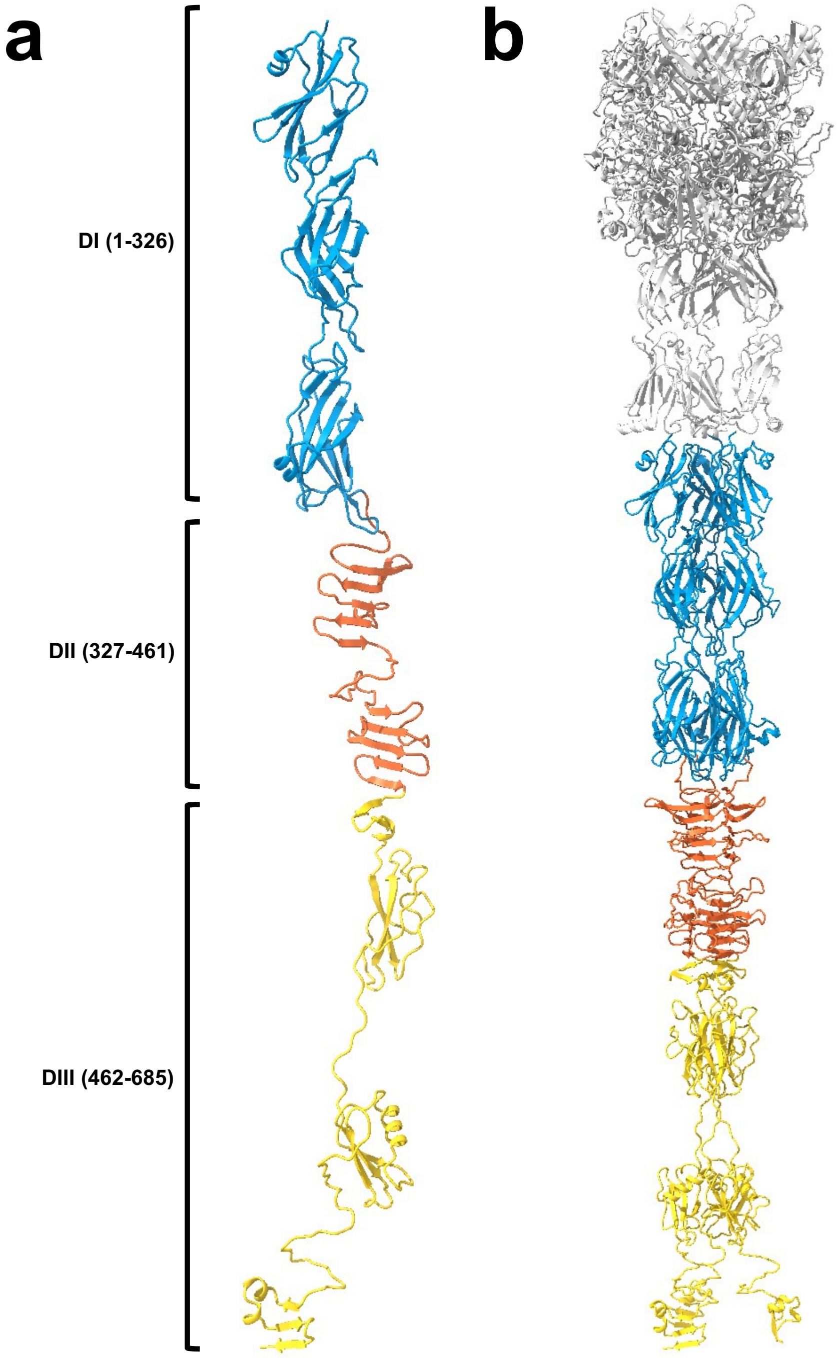


**Supplementary Figure 23. Model for the central tail fiber protein. a**, Structure of a CFP monomer. **b**, Structure of the CFP trimer bound to the bottom part of the TTHP trimer (grey).

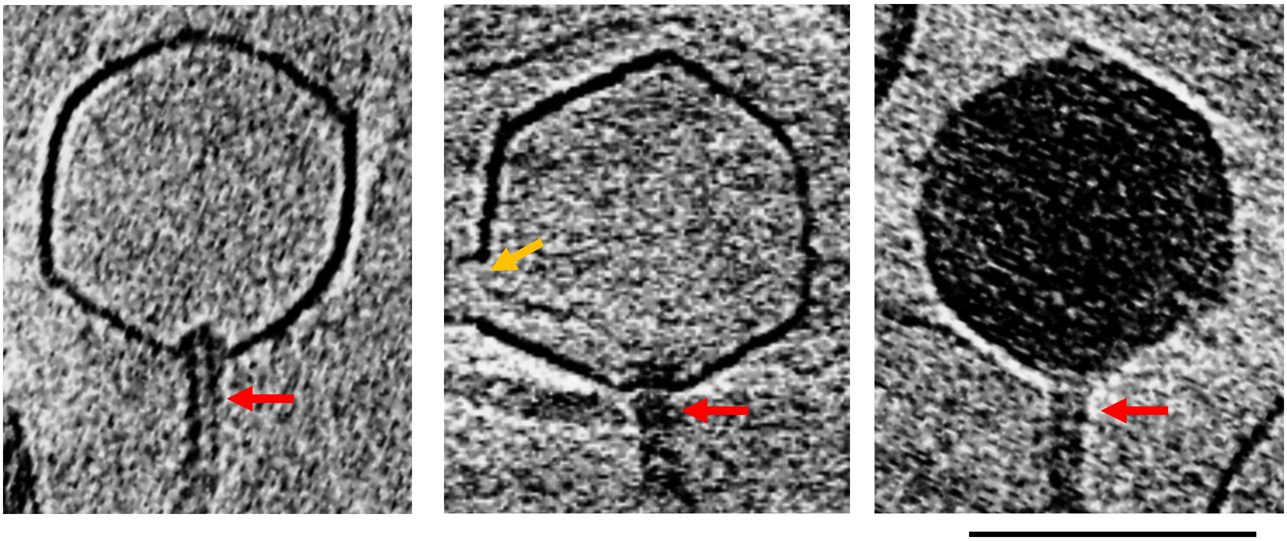

**Supplementary Figure 24. Tomograms of DT57C viruses in different stages.** Tomograms of viruses in three different states were collected: empty virus with intact capsid (left), empty virus with a broken capsid (middle) and full virus with intact capsid (right). A total of 9 tomograms containing 63 virions were collected, of which 50 were full viruses with intact capsids, 12 were empty viruses with intact capsids and 1 was an empty virus with a broken capsid. Red arrows highlight the tail of each virus. The tail of the empty virus with an intact capsid displays a hollow tail, which features two parallel layers of density with no inner density between them. This is due to the ejection of both DNA and TMP through the tail. The tail of the full virus is also full since it contains the TMP, and displays internal density between the outer walls of the tail tube. The virus with a broken capsid displays an empty capsid, since the DNA was released through the breakage point (yellow arrow). Nevertheless, in spite of the DNA being released from the capsid, the TMP remains within the tail tube, as evidenced by the fact that the tail displays internal density, similar to that observed in the tail of the full, intact virus. The scale bar represents a distance of 800 Å.

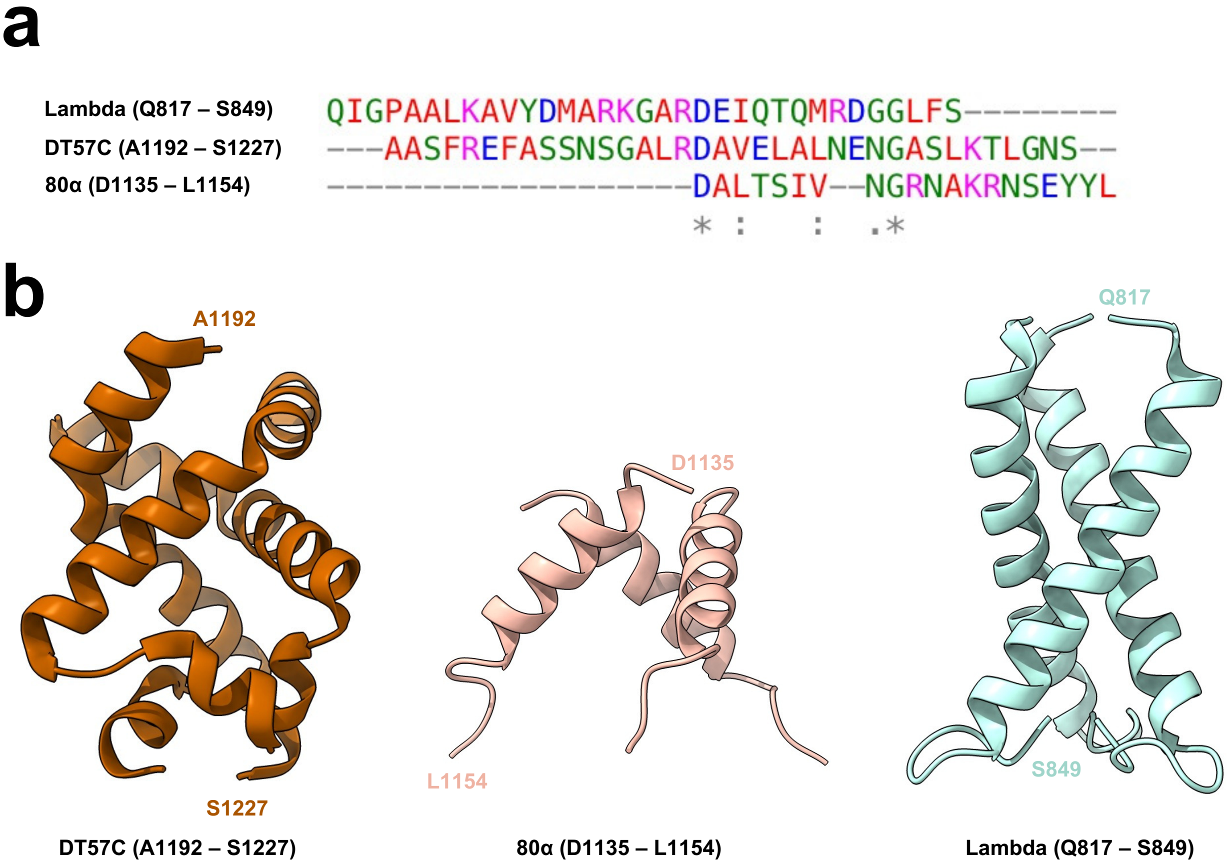


**Supplementary Figure 25. Comparison of TMP C-terminal regions.** The C-terminal fragments of the TMP of phages DT57C, 80α (PDB code 6V8I) and λ (8IYK) are compared. **a**, Multiple sequence alignment. No conserved sequence motifs were identified. **b**, Structural comparison. In all cases, the TMP C-terminal fragment adopts a trimeric, α-helical coiled-coil conformation.

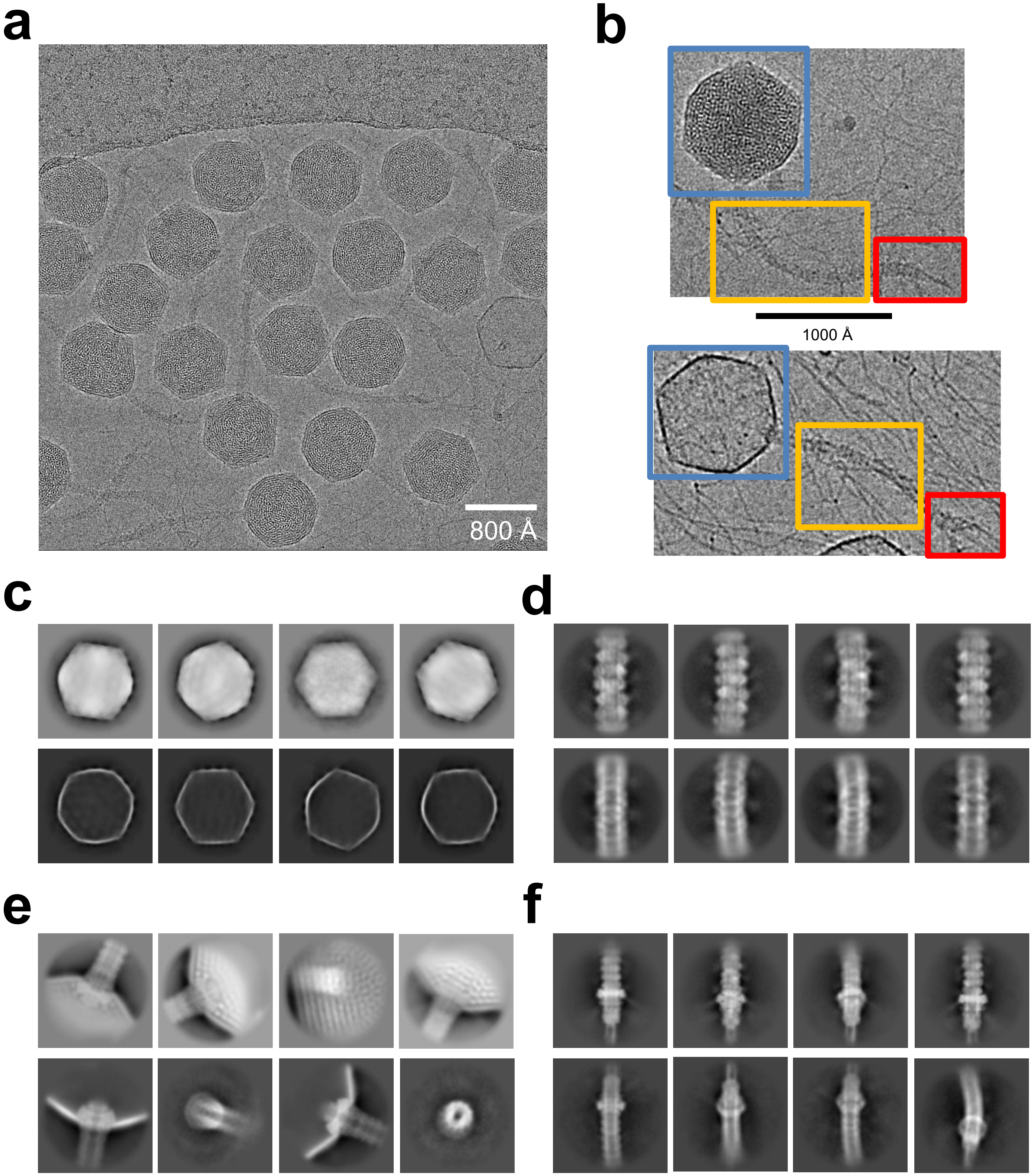


**Supplementary Figure 26. Overview of cryo-EM data.** **a**, Example micrograph (out of a total of 34,852). While the majority of particles corresponded to viruses filled with genetic material, a minority of empty viruses were also found. **b**, Example particles with different regions highlighted (blue – capsid, yellow – tail, red – tail tip). **c – f**, Representative 2D classes of the different regions of the virus: capsid (**c**), tail tube (**d**), neck (**e**) and tail tip (**f**). For each region, 2D classes of both the full (top) and empty (bottom) viruses are shown.

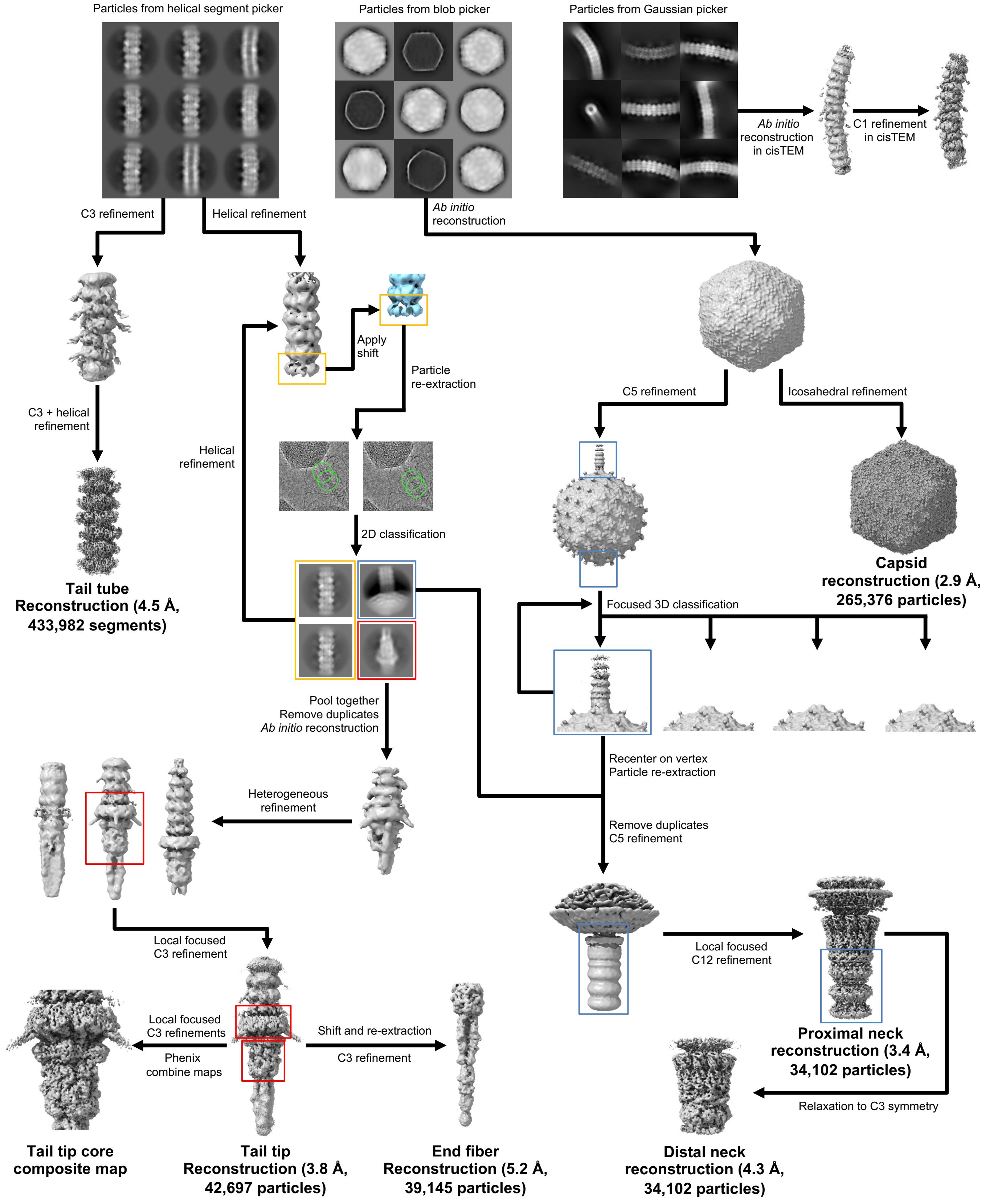


**Supplementary Figure 27. Cryo-EM data processing workflow.** A detailed description of the procedure can be found in the Materials and Methods section. The procedure was carried independently for particles corresponding to full and empty viruses. Shown resolution values indicate the resolution of the reconstruction of the corresponding region for the full viruses.

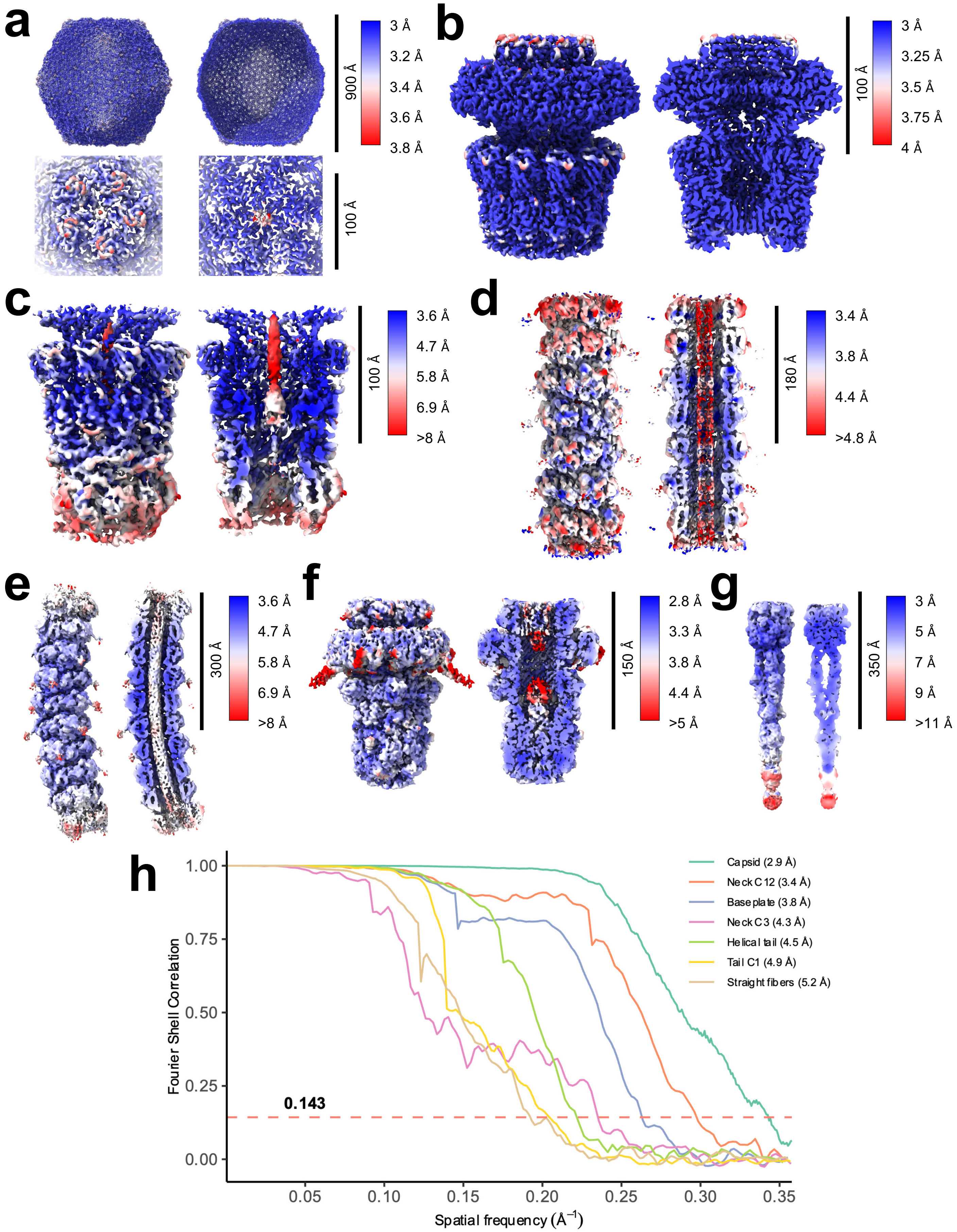


**Supplementary Figure 28. Quality of cryo-EM reconstructions. a-g**, Local resolution maps of the capsid (**a**), neck C12 (**b**), neck C3 (**c**), helical tail segment (**d**), bent tail C1 (**e**), baseplate (**f**) and straight fibers (**g**) full virus reconstructions. **h**, Corrected FSC curves of the reconstructions. Source data for panel **h** are provided as a Source Data file.

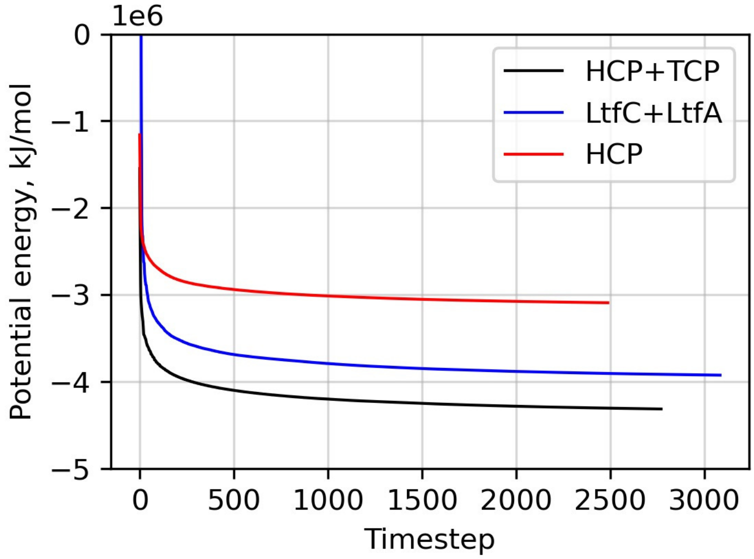


**Supplementary Figure 29. Changes in the potential energy during the energy minimization of the three simulated systems.** Source data are provided as a Source Data file.

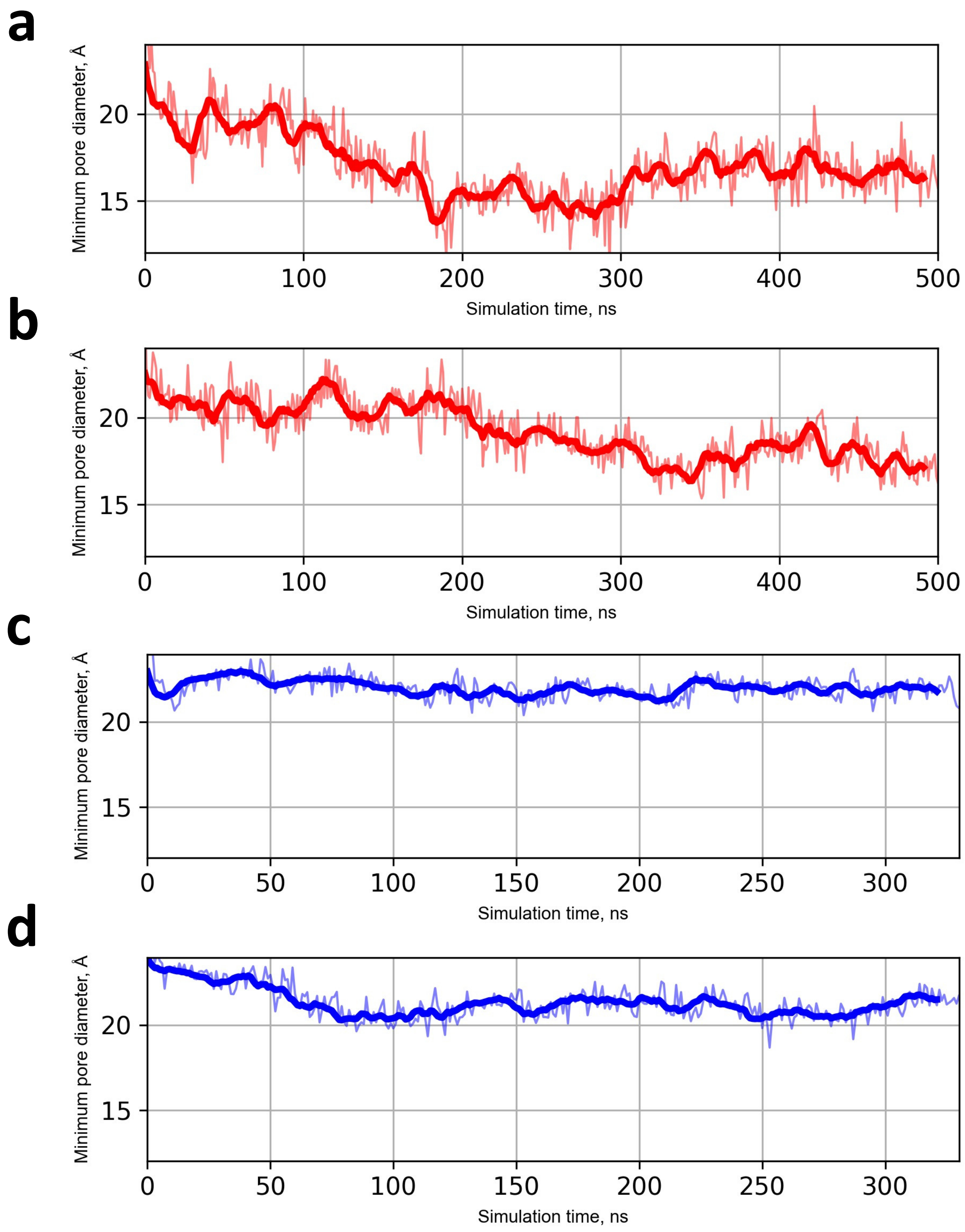


**Supplementary Figure 30. Evolution of the minimum pore diameter during the replicate MD simulations. a-b**, central pore of the HCP ring in the absence of TCP. **c-d**, central pore of the HCP ring in the presence of TCP.

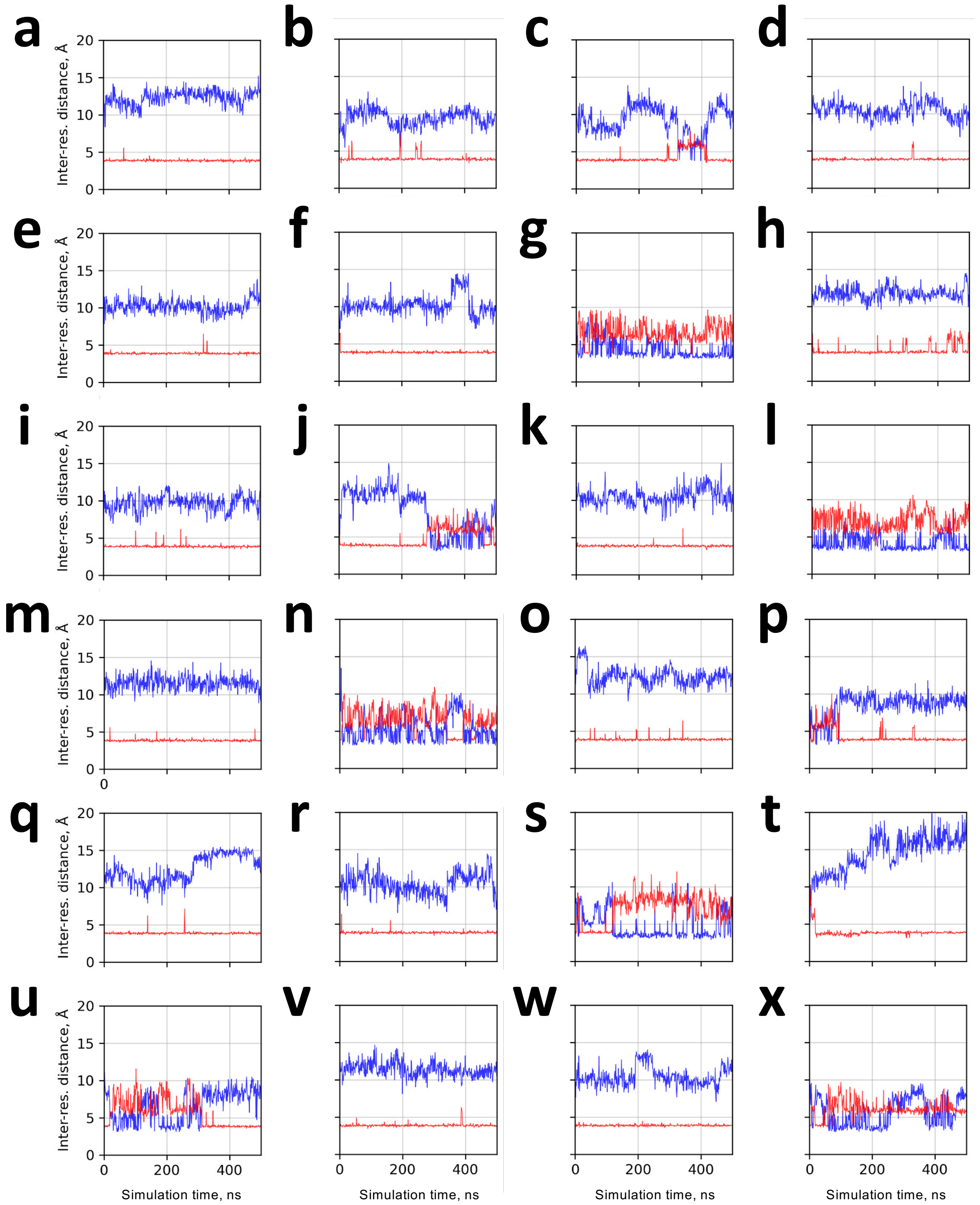


**Supplementary Figure 31. Contacts in HCP protomers in the absence of the TCP ring in two replicate MD simulations. a-l**, Inter-residue distances between D135-K140 of each HCP protomer (red) and K140-E145 of neighboring HPC protomers (blue) in the simulation replicate #2 of the HCP ring. **m-x**, Inter-residue distances between D135-K140 of each HCP protomer (red) and K140-E145 of neighboring HPC protomers (blue) in the simulation replicate #3 of the HCP ring. Source data are provided as a Source Data file.

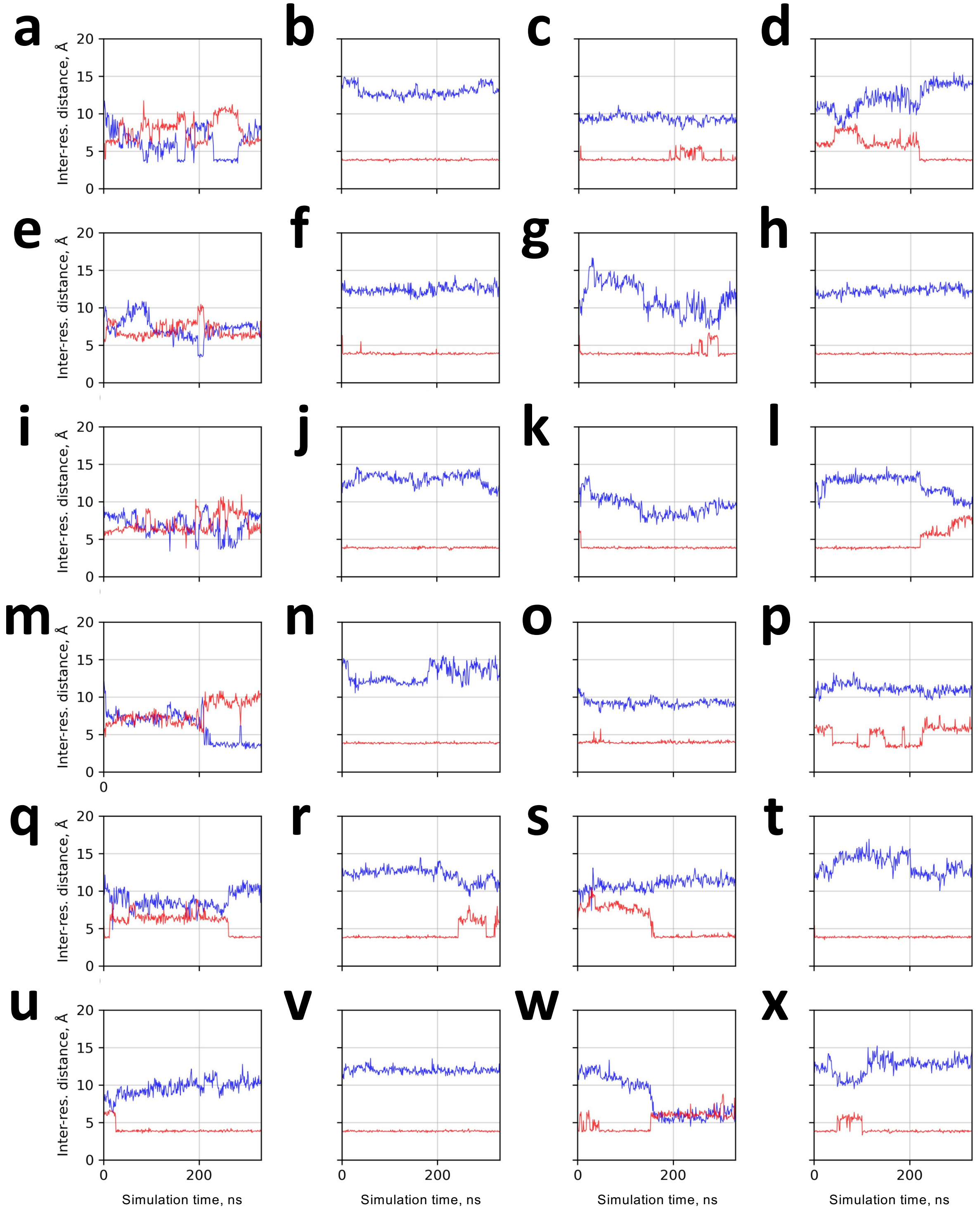


**Supplementary Figure 32. Contacts in HCP protomers in the presence of the TCP ring in two replicate MD simulations**. **a-l**, Inter-residue distances between D135-K140 of each HCP protomer (red) and K140-E145 of neighboring HPC protomers (blue) in the simulation replicate #2 of the HCP ring. **m-x**, Inter-residue distances between D135-K140 of each HCP protomer (red) and K140-E145 of neighboring HPC protomers (blue) in the simulation replicate #3 of the HCP ring. Source data are provided as a Source Data file.

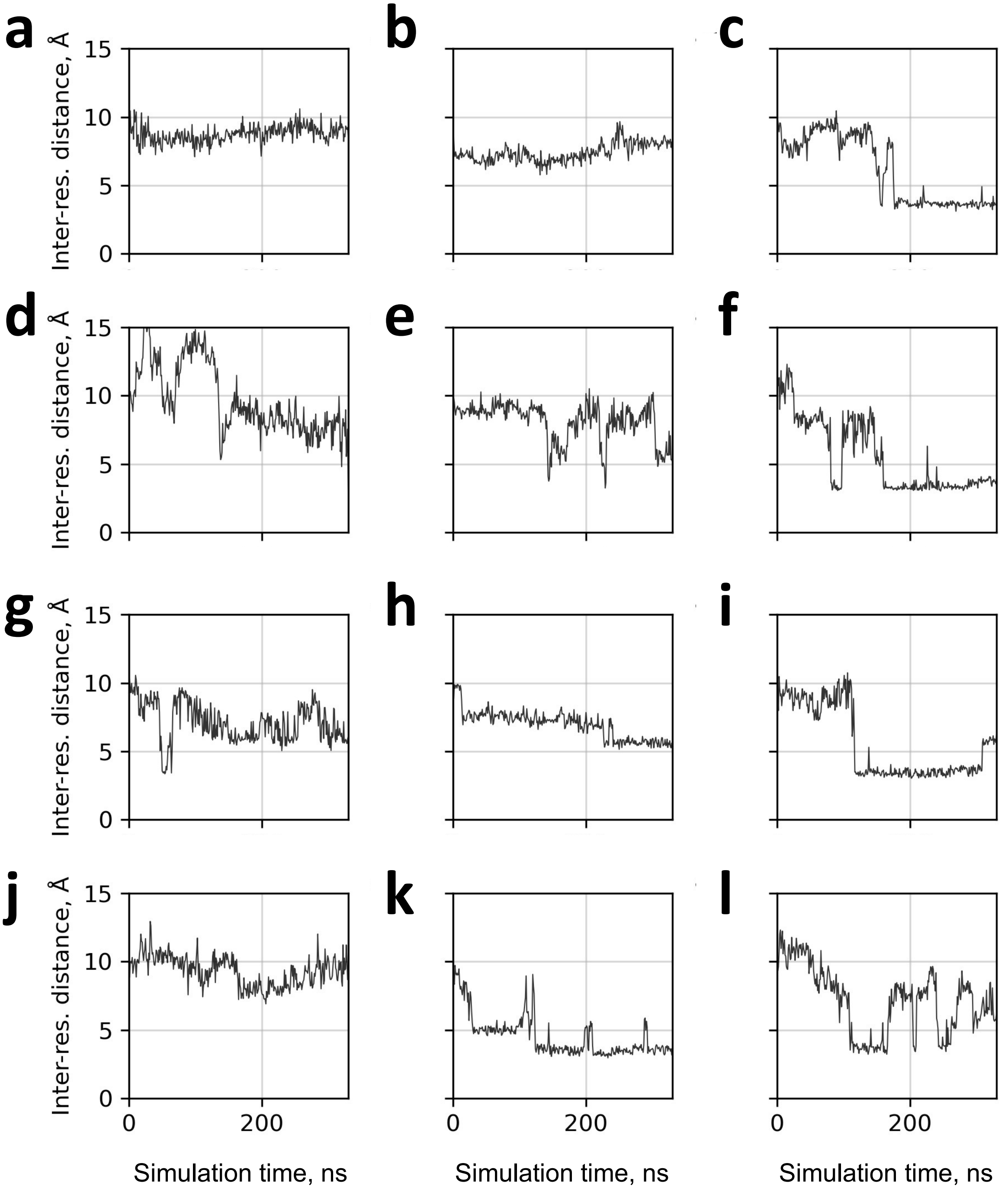


**Supplementary Figure 33. Contacts between HCP and TCP protomers in two replicate MD simulations. a-f**, Inter-residue distances between E145 of HCP and K41 of adjacent TCP protomers in simulation replicate #2. **g-l**, Inter-residue distances between E145 of HCP and K41 of adjacent TCP protomers in simulation replicate #3. Due to the mismatch in the number of protomers in the HCP and TCP rings, only six such contacts can potentially form. Source data are provided as a Source Data file.

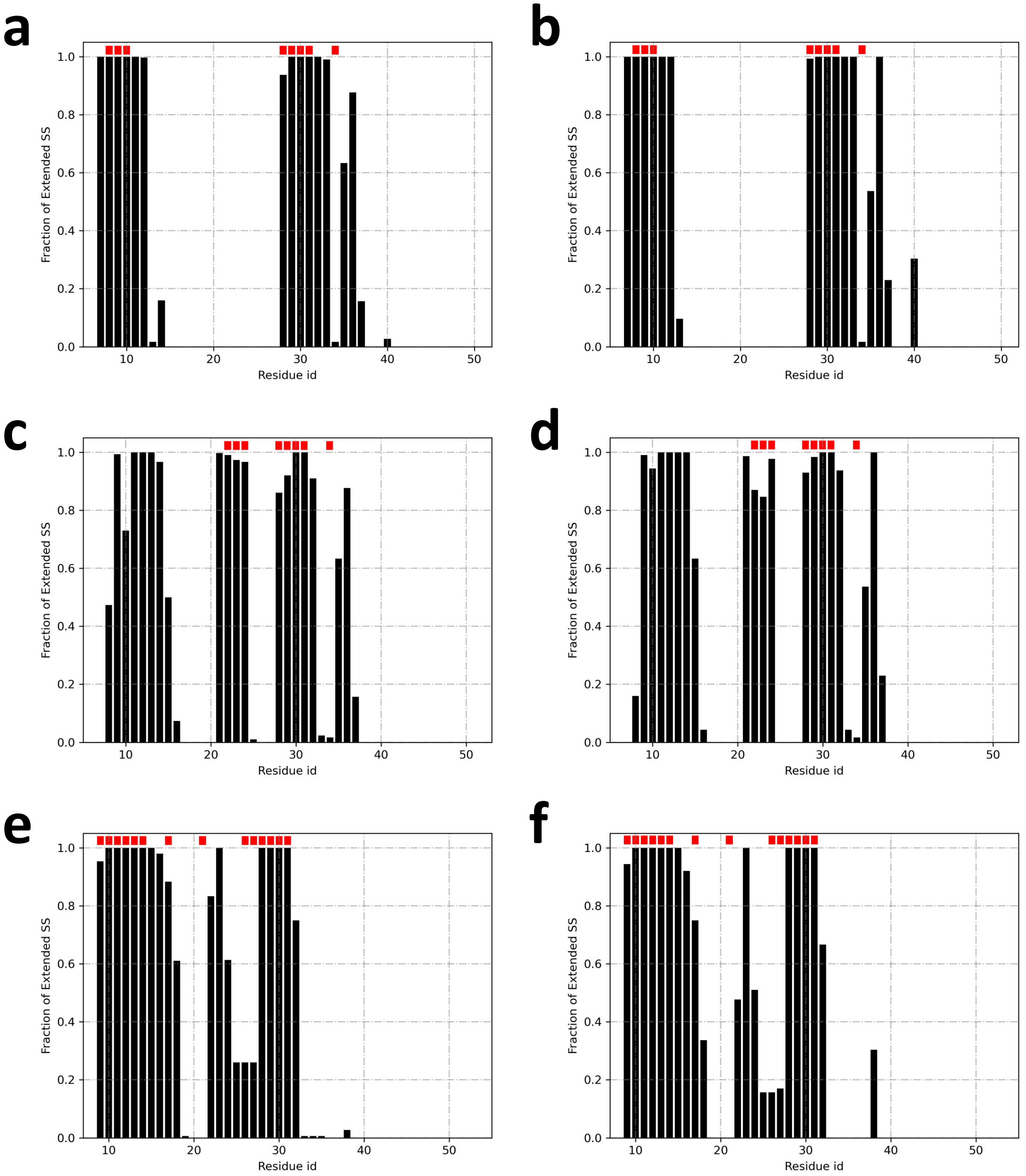


**Supplementary Figure 34. Propensity of the LtfA residues to form extended secondary structure (β-sheets or isolated β-bridges) in two replicate MD simulations. a-c**, the three different monomers of the LtfA trimer in simulation replicate #2. **d-e**, as in **a-c** but for simulation replicate #3. Values for each monomer are averaged over three LtfA trimers. The red bars at the top of the histograms indicate the residues possessing the extended conformation in the initial model. Source data are provided as a Source Data file.

**Supplementary Table 1. Data collection and model building statistics.** Data are provided for the four different regions of the virus that were reconstructed (tail tip, neck, tail and capsid).

| | Tail tip | Neck | Helical tail | Capsid |
|---|---|---|---|---|
| **Data collection and processing** | | | | |
| Microscope and detector | Titan Krios/Falcon 3EC | Titan Krios/Falcon 3EC | Titan Krios/Falcon 3EC | Titan Krios/Falcon 3EC |
| Accelerating voltage (kV) | 300 | 300 | 300 | 300 |
| Nominal magnification | 78,000 | 78,000 | 78,000 | 78,000 |
| Total exposure (e/Å$^2$) | 67 | 67 | 67 | 67 |
| Defocus range (μm) | -0.7 to -2.0 | -0.7 to -2.0 | -0.7 to -2.0 | -0.7 to -2.0 |
| Pixel size (Å) | 1.387 | 1.387 | 1.387 | 1.387 |
| Final number of particles | | | | |
| Map resolution at 0.143 FSC criterion (Å) | 3.8 | 3.4 (C12 part), 4.3 (C3 part), 7.9 (TMP part) | 4.5 | 2.9 |
| Symmetries imposed | C3 | C12 (proximal part), C3 (distal and TMP parts) | C3, Helical (rise 41.84 Å, twist 39.51 º) | Icosahedral |
| **Model refinement** | | | | |
| Initial models | Alphafold2, ModelAngelo | Alphafold2, ModelAngelo | Alphafold2 | ModelAngelo |
| Model resolution $d_{model}$ (Å) | 4.1 | 3.8 | 4.6 | |
| Model map CC | 0.81 | 0.78 | 0.82 | 0.83 |
| Model composition | | | | |
| Non-hydrogen atoms | 66606 | 60711 | 21312 | 1783560 |
| Protein residues | 8520 | 7656 | 2796 | 230340 |
| Ligands | 0 | 0 | 0 | 0 |
| R.m.s. deviations | | | | |
| Bond lengths (Å) | 0.002 | 0.007 | 0.003 | 0.005 |
| Bond angles (°) | 0.518 | 1.122 | 0.685 | 0.568 |
| Validation | | | | |
| MolProbity score | 1.72 | 1.4 | 1.98 | 1.86 |
| Clashscore | 8.47 | 5.2 | 10.67 | 8.68 |
| Rotamer outliers (%) | 0.00 | 0.00 | 0.00 | 0.13 |
| CaBLAM outliers (%) | 1.58 | 1.81 | 2.16 | 2.09 |
| Ramachandran plot | | | | |
| Favored (%) | 96.09 | 97.36 | 93.32 | 94.20 |
| Allowed (%) | 3.59 | 2.64 | 6.47 | 5.80 |
| Outliers (%) | 0.32 | 0.00 | 0.22 | 0.00 |
| Rama-Z | | | | |
| whole | 0.24 | -1.42 | -0.82 | -0.09 |
| helix | 1.07 | -0.79 | 1.54 | 0.91 |
| sheet | 0.86 | -0.07 | 0.45 | 0.08 |
| loop | -0.51 | -1.22 | -1.75 | -0.43 |

**Supplementary Table 2. Regions of each structural protein present in the reconstructions.** Residue ranges covering ordered regions and regions with weak density, as well as the total length, are shown for each structural protein present in the models.

| | Total length | Ordered regions | Regions with weak density |
|---|---|---|---|
| **MCP** | 458 (1-160 cleaved) | 161-364, 374-458 | – |
| **PrtP** | 405 | 11-378 | – |
| **HCP** | 170 | 1-170 | – |
| **TCP** | 161 | 2-161 | – |
| **TMP** | 1227 | 1-80, 1192-1227 | – |
| **TTP** | 468 | 3-376 | 377-468 |
| **LtfC** | 140 | 2-140 | – |
| **TTMP** | 300 | 2-297 | – |
| **LtfA** | 1076 | 6-51 | – |
| **Dit** | 204 | 1-204 | – |
| **TTHP** | 949 | 1-747, 757-918, 929-949 | – |
| **CFP** | 685 | 1-478 | 479-685 |
