# Supplementary material for: Nearly complete structure of bacteriophage DT57C reveals architecture of head-to-tail interface and lateral tail fibers
Source: Nat Commun. 2023 Dec 11;14:8205. doi: 10.1038/s41467-023-43824-9 (PMC10713586; doi:10.1038/s41467-023-43824-9)
Supplement: Supplementary file 3 — Description of additional supplementary files [file 41467_2023_43824_MOESM3_ESM.pdf]

Description of additional supplementary files

Supplementary Movie 1. Molecular dynamics trajectory of the HCP ring, which revealed that the β-hairpins of the DI of HCP are prone to large-scale fluctuations resulting in conformations partially occluding the central pore. Proteins are colored according to their secondary structure (αhelices - red, β-sheets - cyan, unstructured coil - magenta).

Supplementary Movie 2. Switching between two alternative salt bridges, D135- K140 and K140-E145, which stabilize the open and closed conformations of the central HCP pore, respectively, as observed in the molecular dynamics simulation of the HCP ring. The relevant amino acid residues in two neighboring protomers of the HCP ring (colored cyan and magenta) are shown.

Supplementary Movie 3. Example tomogram of DT57C.

Supplementary Movie 4. Animation of the collective motion corresponding to the first principal component derived from the MD simulation of the LtfA-LtfC-TTMP complex.
