# Supplementary material for: Nearly complete structure of bacteriophage DT57C reveals architecture of head-to-tail interface and lateral tail fibers
Source: Nat Commun. 2023 Dec 11;14:8205. doi: 10.1038/s41467-023-43824-9 (PMC10713586; doi:10.1038/s41467-023-43824-9)
Supplement: Supplementary file 8 — Reporting Summary [file 41467_2023_43824_MOESM8_ESM.pdf]

nature portfolio

Corresponding author(s): Olga S. Sokolova, Andrey V. Letarov, Matthias Wolf

Last updated by author(s): Nov 13, 2023

Reporting Summary

Nature Portfolio wishes to improve the reproducibility of the work that we publish. This form provides structure for consistency and transparency in reporting. For further information on Nature Portfolio policies, see our Editorial Policies and the Editorial Policy Checklist.

Statistics

For all statistical analyses, confirm that the following items are present in the figure legend, table legend, main text, or Methods section.

n/a	Confirmed	
☒	☐	The exact sample size (*n*) for each experimental group/condition, given as a discrete number and unit of measurement
☒	☐	A statement on whether measurements were taken from distinct samples or whether the same sample was measured repeatedly
☒	☐	The statistical test(s) used AND whether they are one- or two-sided<br>*Only common tests should be described solely by name; describe more complex techniques in the Methods section.*
☒	☐	A description of all covariates tested
☒	☐	A description of any assumptions or corrections, such as tests of normality and adjustment for multiple comparisons
☐	☒	A full description of the statistical parameters including central tendency (e.g. means) or other basic estimates (e.g. regression coefficient) AND variation (e.g. standard deviation) or associated estimates of uncertainty (e.g. confidence intervals)
☒	☐	For null hypothesis testing, the test statistic (e.g. *F*, *t*, *r*) with confidence intervals, effect sizes, degrees of freedom and *P* value noted<br>*Give P values as exact values whenever suitable.*
☒	☐	For Bayesian analysis, information on the choice of priors and Markov chain Monte Carlo settings
☒	☐	For hierarchical and complex designs, identification of the appropriate level for tests and full reporting of outcomes
☒	☐	Estimates of effect sizes (e.g. Cohen's *d*, Pearson's *r*), indicating how they were calculated

Our web collection on statistics for biologists contains articles on many of the points above.

Software and code

Policy information about availability of computer code

Data collection	Cryo-EM data was collected with EPU 2.12.
Data analysis	Cryo-EM data was processed with cryoSPARC 4.1.0 and RELION 3.1. Initial atomic models were obtained with ModelAngelo 0.2.4 or AlphaFold 2.0. Atomic models were manually built with Coot 0.9.4 and ISOLDE 1.6.0, and refined with phenix 1.19.2. Structures were visualized with ChimeraX 1.5. Minimization and equilibration of initial models for molecular dynamics simulations were performed with CHARMM 36, and molecular dynamics simulations were performed with GROMACS 2020.3. Secondary structure analysis was performed with MDTraj 1.9.7. Principal component analysis was performed with ProDy 2.4.0. Electrostatic potential calculations were performed with APBS 3.4.1. Inner pore diameter of the HCP ring was calculated with HOLE 2.0.

For manuscripts utilizing custom algorithms or software that are central to the research but not yet described in published literature, software must be made available to editors and reviewers. We strongly encourage code deposition in a community repository (e.g. GitHub). See the Nature Portfolio guidelines for submitting code & software for further information.

Data

Policy information about availability of data

All manuscripts must include a data availability statement. This statement should provide the following information, where applicable:

- Accession codes, unique identifiers, or web links for publicly available datasets
- A description of any restrictions on data availability
- For clinical datasets or third party data, please ensure that the statement adheres to our policy

The atomic models generated in this study have been deposited in the PDB under accession codes 8HQZ [http://doi.org/10.2210/pdb8HQZ/pdb] (baseplate), 8HRG [http://doi.org/10.2210/pdb8HRG/pdb] (helical tail tube), 8HRE [http://doi.org/10.2210/pdb8HRE/pdb] (straight tail fibers), 8HQO [http://doi.org/10.2210/pdb8HQI/pdb] (neck) and 8HO3 [http://doi.org/10.2210/pdb8HO3/pdb] (capsid). Electron density maps have been deposited in the EMDB under accession codes 34955 [https://www.ebi.ac.uk/pdbe/entry/emdb/EMD-34955] (baseplate), 34972 [https://www.ebi.ac.uk/pdbe/entry/emdb/EMD-34972] (helical tail tube), 34973 [https://www.ebi.ac.uk/pdbe/entry/emdb/EMD-34973] (curved tail segment), 34968 [https://www.ebi.ac.uk/pdbe/entry/emdb/EMD-34968] (straight tail fibers), 34952 [https://www.ebi.ac.uk/pdbe/entry/emdb/EMD-34952] (neck) and 34920 [https://www.ebi.ac.uk/pdbe/entry/emdb/EMD-34920] (capsid). Tomograms have been deposited in the EMDB under accession codes 37518 [https://www.ebi.ac.uk/pdbe/entry/emdb/EMD-37518], 37519 [https://www.ebi.ac.uk/pdbe/entry/emdb/EMD-37519], 37521 [https://www.ebi.ac.uk/pdbe/entry/emdb/EMD-37521], 37531 [https://www.ebi.ac.uk/pdbe/entry/emdb/EMD-37531], 37534 [https://www.ebi.ac.uk/pdbe/entry/emdb/EMD-37534], 37536 [https://www.ebi.ac.uk/pdbe/entry/emdb/EMD-37536], 37539 [https://www.ebi.ac.uk/pdbe/entry/emdb/EMD-37539], 37543 [https://www.ebi.ac.uk/pdbe/entry/emdb/EMD-37543] and 37544 [https://www.ebi.ac.uk/pdbe/entry/emdb/EMD-37544]. Initial structures for molecular dynamics simulations along with the resulting trajectories have been deposited in Zenodo with identifier [https://doi.org/10.5281/zenodo.8343960]. The structures of the C-terminal fragment of the TMP of other phages are available at the PDB under accession codes 6V8I [http://doi.org/10.2210/pdb6V8I/pdb] (80α) and 8IYK [http://doi.org/10.2210/pdb8IYK/pdb] (λ). Source data are provided with this paper.

Research involving human participants, their data, or biological material

Policy information about studies with human participants or human data. See also policy information about sex, gender (identity/presentation), and sexual orientation and race, ethnicity and racism.

Reporting on sex and gender	N/A
Reporting on race, ethnicity, or other socially relevant groupings	N/A
Population characteristics	N/A
Recruitment	N/A
Ethics oversight	N/A

Note that full information on the approval of the study protocol must also be provided in the manuscript.

Field-specific reporting

Please select the one below that is the best fit for your research. If you are not sure, read the appropriate sections before making your selection.

☒ Life sciences ☐ Behavioural & social sciences ☐ Ecological, evolutionary & environmental sciences

For a reference copy of the document with all sections, see nature.com/documents/nr-reporting-summary-flat.pdf

Life sciences study design

All studies must disclose on these points even when the disclosure is negative.

Sample size	No statistical methods were used to predetermine sample size except sufficient images were collected to ensure adequate reconstructions can be obtained. A total of 34,852 micrographs were collected since this was estimated to be enough to obtain high-resolution reconstructions.
Data exclusions	No data were initially excluded from the analysis although final reconstructions were obtained from subset of the data as detailed in Methods section.
Replication	Findings were reproducible. Phage infectivity was confirmed as phages were propagated before purification as described in (Golomidova et al., 2015). Thousands of micrographs with this sample were consistent in quality.
Randomization	All data were used in an unbiased way for data analysis and image reconstruction. Final quality control (such as resolution estimation) were conducted with gold-standard FSC by randomly separating data into two halves and refining them independently.
Blinding	No blinding was performed as it is not relevant to this type of study. This is due to the fact that the goal of cryo-EM studies is to determine the structure of a given target sample, and therefore sample information must be known a priori.

Reporting for specific materials, systems and methods

We require information from authors about some types of materials, experimental systems and methods used in many studies. Here, indicate whether each material, system or method listed is relevant to your study. If you are not sure if a list item applies to your research, read the appropriate section before selecting a response.

Materials & experimental systems

n/a	Involved in the study	
☒	☐	Antibodies
☒	☐	Eukaryotic cell lines
☒	☐	Palaeontology and archaeology
☒	☐	Animals and other organisms
☒	☐	Clinical data
☒	☐	Dual use research of concern
☒	☐	Plants

Methods

n/a	Involved in the study	
☒	☐	ChIP-seq
☒	☐	Flow cytometry
☒	☐	MRI-based neuroimaging
